# Supplementary material for: Revealing proteome-level functional redundancy in the human gut microbiome using ultra-deep metaproteomics
Source: Nat Commun. 2023 Jun 10;14:3428. doi: 10.1038/s41467-023-39149-2 (PMC10257714; doi:10.1038/s41467-023-39149-2)
Supplement: Supplementary file 1 — Supplementary information [file 41467_2023_39149_MOESM1_ESM.pdf]

Supplementary Figures

Supplementary Notes

Supplementary Tables

Revealing Proteome-Level Functional Redundancy in the Human Gut Microbiome using Ultra-deep Metaproteomics

Leyuan Li[1,2,†], Tong Wang[3,†], Zhibin Ning[2], Xu Zhang[2], James Butcher[4], Joeselle M. Serrana[2], Caitlin M.A. Simopoulos[2], Janice Mayne[2], Alain Stintzi[4], David R. Mack[5], Yang-Yu Liu[3,6,*], Daniel Figeys[2,*]

Affiliations

[1]*State Key Laboratory of Proteomics, Beijing Proteome Research Center, National Center for Protein Sciences (Beijing), Beijing Institute of Lifeomics, Beijing 102206, China*

[2]*School of Pharmaceutical Sciences and Ottawa Institute of Systems Biology, Faculty of Medicine, University of Ottawa, Ottawa, ON K1H 8M5, Canada*

[3]*Channing Division of Network Medicine, Department of Medicine, Brigham and Women's Hospital and Harvard Medical School, Boston, MA 02115, USA*

[4]*Department of Biochemistry, Microbiology and Immunology, Faculty of Medicine, University of Ottawa, Ottawa, ON K1H 8M5, Canada*

[5]*Department of Paediatrics, Faculty of Medicine, University of Ottawa and Children's Hospital of Eastern Ontario Inflammatory Bowel Disease Centre and Research Institute, Ottawa, ON K1H 8L1, Canada*

[6]*Center for Artificial Intelligence and Modeling, The Carl R. Woese Institute for Genomic Biology, University of Illinois at Urbana-Champaign, Urbana, IL 61801, USA.*

*Correspondence, D.F.: dfigeys@uottawa.ca; Y-Y.L.: yyl@channing.harvard.edu

[†] Those authors contributed equally.

Supplementary Figures

Figure S1. Calculation of FR_p and nFR_p

Figure S2. Construction of a reference GCN and difficulty of constructing a reference PCN

Figure S3. *In silico* community demonstrates the sensitivity of the nFR_p metrics. A

Figure S4. Comparison of feature dimensions in GCN and PCN

Figure S5. Tripartite plot showing taxonomic and functional relationships between GCN and PCN for individual microbiome sample HM455

Figure S5. Tripartite plot showing taxonomic and functional relationships between GCN and PCN for individual microbiome sample HM466

Figure S7. Tripartite plot showing taxonomic and functional relationships between GCN and PCN for individual microbiome sample HM503

Figure S8. More simulations performed by altering byproduct fraction and externally supplied nutrient diversity

Figure S9. More simulations performed by altering S:M ratio

Figure S10. Degree distributions of PCNs and GCNs in the other three microbiomes

Figure S11. Degree distributions of the four PCNs generated with IGC-based search

Figure S12. PCNs and corresponding degree distributions in different metaproteomics datasets

Figure S13. Functional redundancy, taxonomic and functional diversity comparisons in the berberine dataset.

Figure S14. Nestedness metric based on Overlap and Decreasing Fill (NODF) of the metaproteomics datasets

Figure S15. Comparison of between-genera dij values across all IBD samples

Figure S16. Distribution of d_{ij} values by compounds in the RapidAIM dataset

Figure S17. Between-genera functional distances in the Berberine dataset

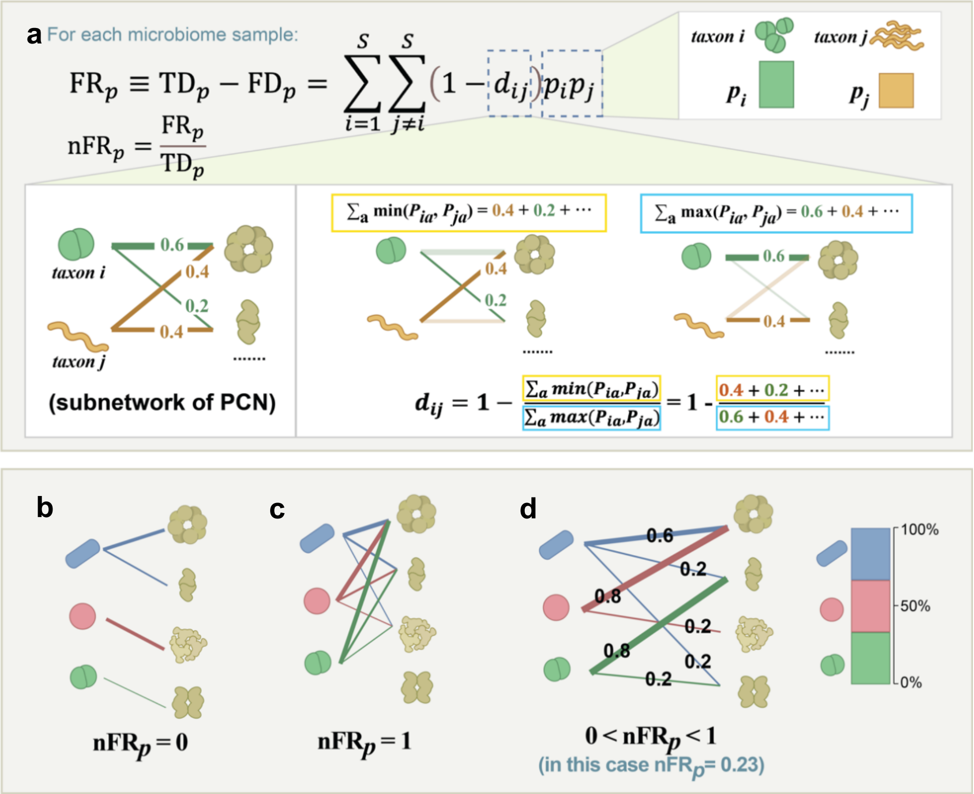

Figure S1. Calculation of FR_p and nFR_p.

a. FR_p is given by the difference between the alpha diversity of taxon-specific protein biomass contributions and the alpha diversity of functional protein abundances. The equation can be transformed into the sum of functional similarity (i.e., 1 - functional distance d_{ij}) between any pair of taxa *i* and *j* multiplied by the biomass contributions of both taxa (p_i and p_j). Calculation of functional distance d_{ij} is based on the subnetwork of taxa *i* and *j* extracted from the sample-specific PCN. d_{ij} is measured by the weighted Jaccard distance between proteomes of taxa *i* and *j*. **b-d**. Examples of nFR_p values demonstrated by a simple conceptual community. **b**. When the expressed proteomes are totally different between different taxa, the nFR_p value equals to 0. **c**. When expressed proteomes are identical between different taxa, the nFR_p value equals to 1. **d**. Under other situations, nFR_p of a microbial community will have a value of between 0 and 1. For example, the conceptual community of (**D**) has a nFR_p value of 0.23. For simplicity, in this conceptual community, we assume that all taxa are equal in biomass. Numbers on the edges represent relative protein abundances of different functions in each taxon.

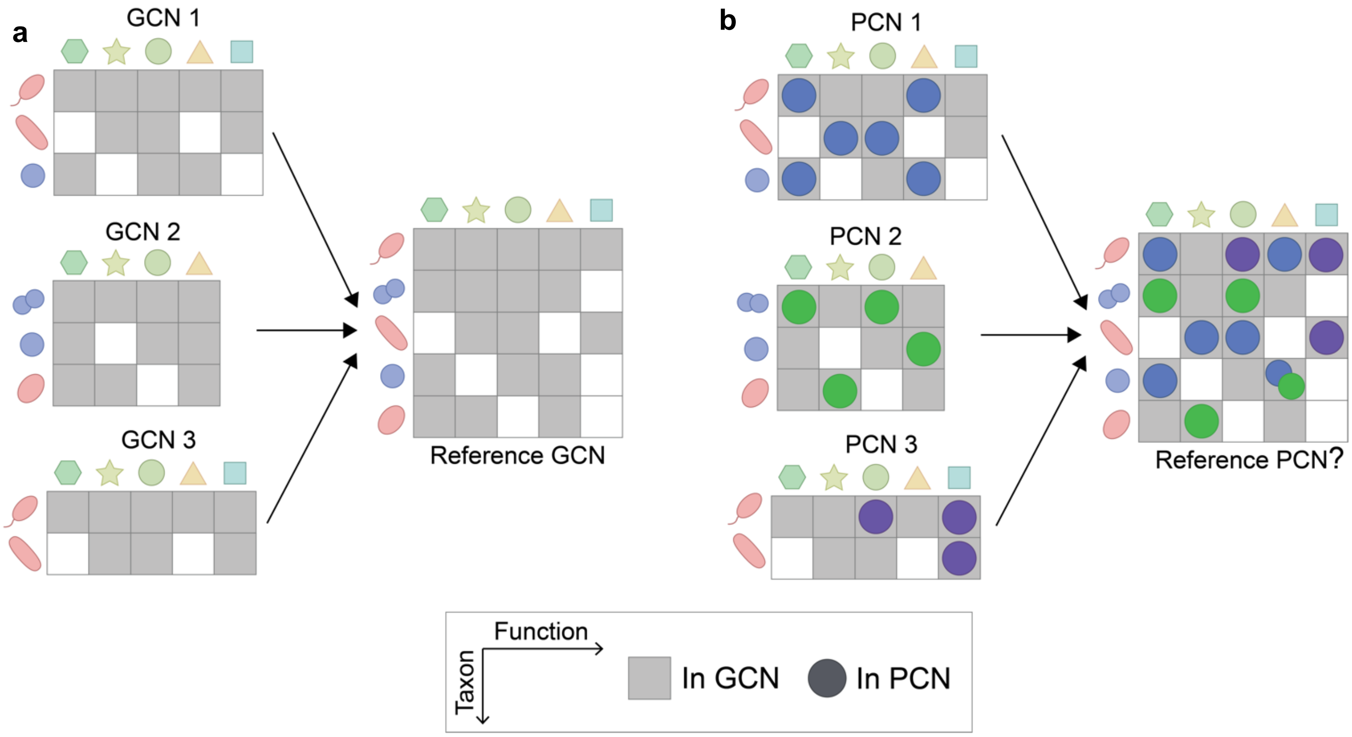

Figure S2. Construction of a reference GCN and difficulty of constructing a reference PCN.

a. Genomic content in each taxon is the same in different microbiome samples, and therefore we can combine different GCNs to form a dataset of multiple GCNs. **b.** Proteomic content of any taxon is sample dependent, and therefore the merged reference PCN is hard to interpret.

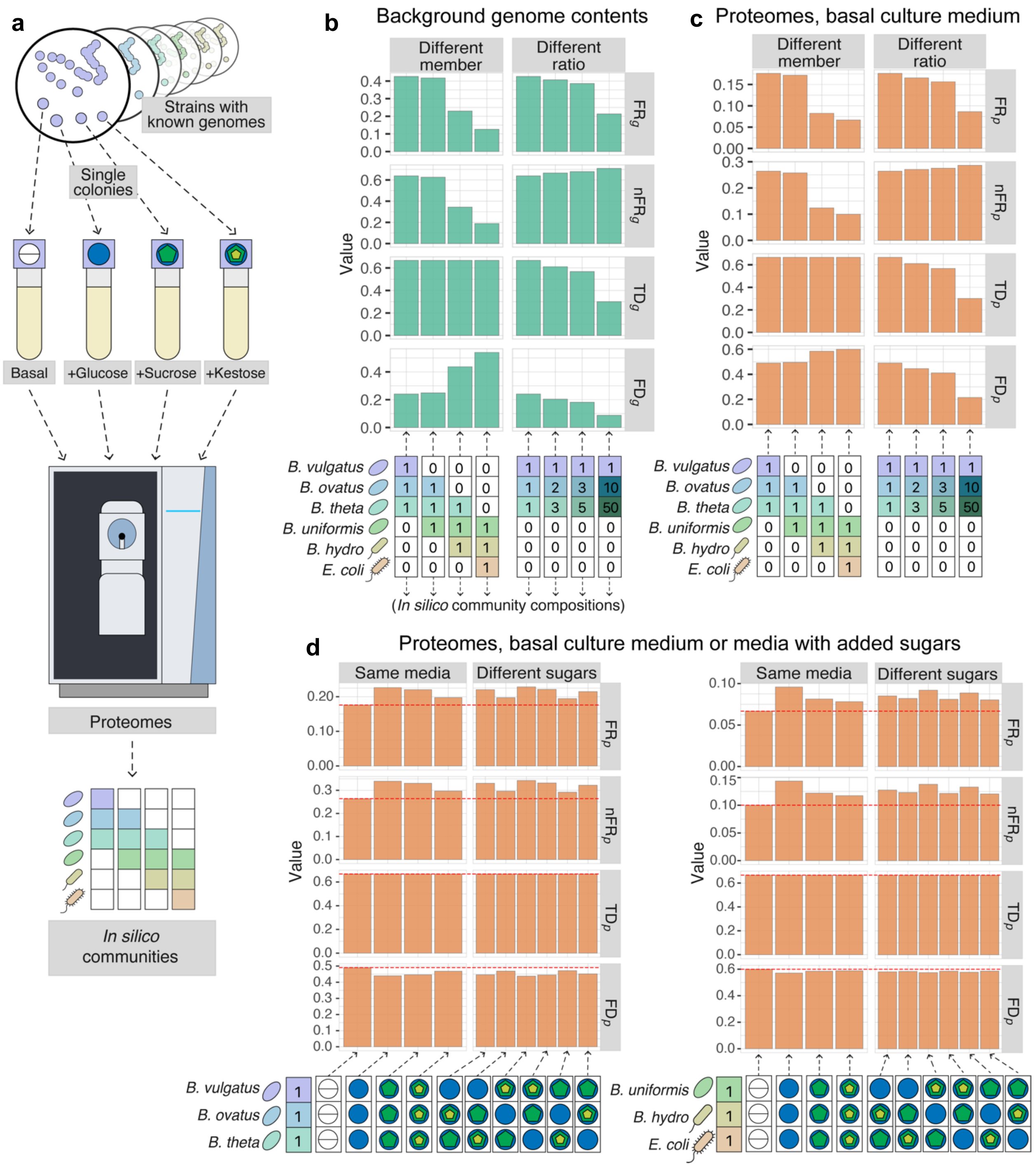

Figure S3. ***In silico*** **community demonstrates the sensitivity of the nFR$_p$ metrics. a.** Illustration of experimental workflow. **b**. Genome-level FR, nFR, TD and FD of different *in silico* communities. **c**. Proteome-level FR, nFR, TD and FD of different *in silico* communities. **d**. Proteome-level FR, nFR, TD and FD of different *in silico* metaproteomes generated using proteomes cultured in different media. Source data are provided as a Source Data file.

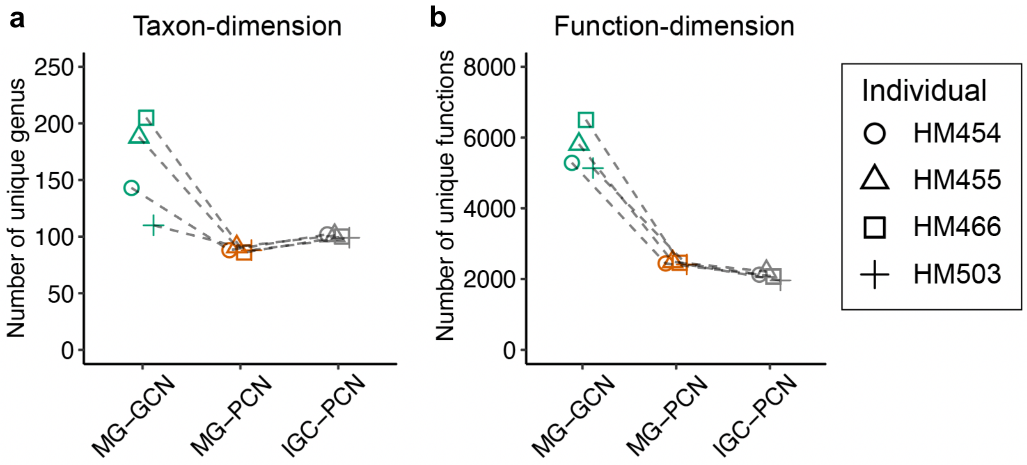

Figure S4. Comparison of feature dimensions in GCN and PCN

Comparison of genus- (**a**) and function-level (**b**) identification between methods. Along the x-axis, MG-GCN group includes GCNs generated with the samples' matched metagenomic sequencing results, and MG-PCN group includes PCNs generated based on search results performed using the matched metagenome databases. IGC-PCN group includes PCNs generated using IGC database and the "protein-peptide bridge" approach. For (**a**), number of genera that had at least 3 unique functions was counted. This was similar to the strategy of using least 3 unique peptides to determine a taxon, as recommended by Jagtap et al. (2015).

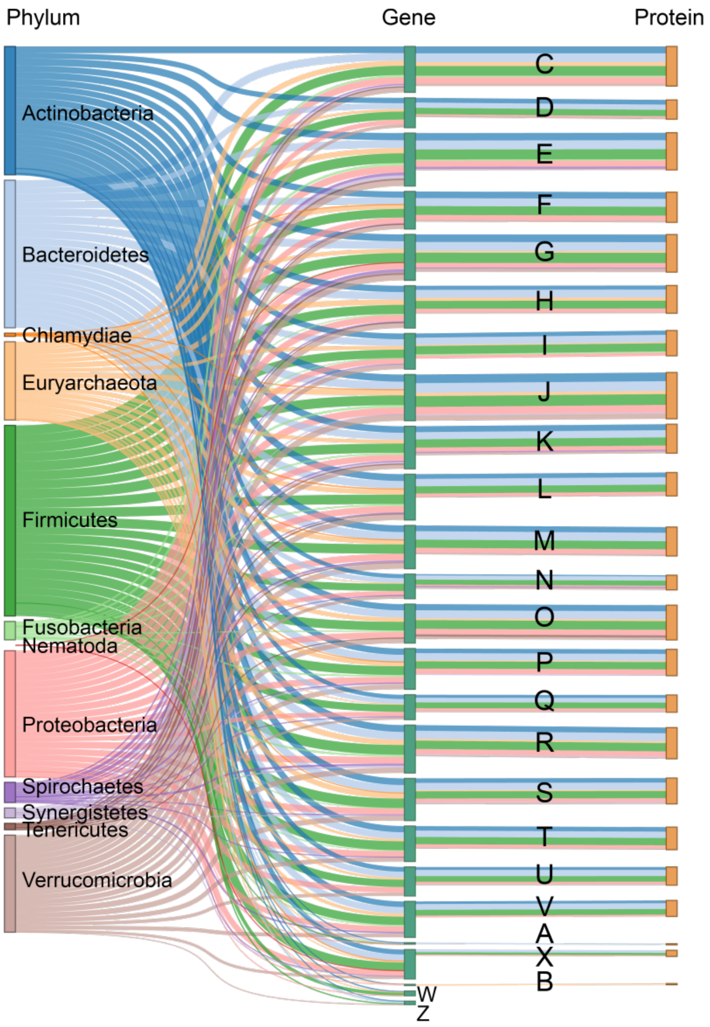

Figure S5. Tripartite plot showing taxonomic and functional relationships between GCN and PCN for individual microbiome sample HM455.

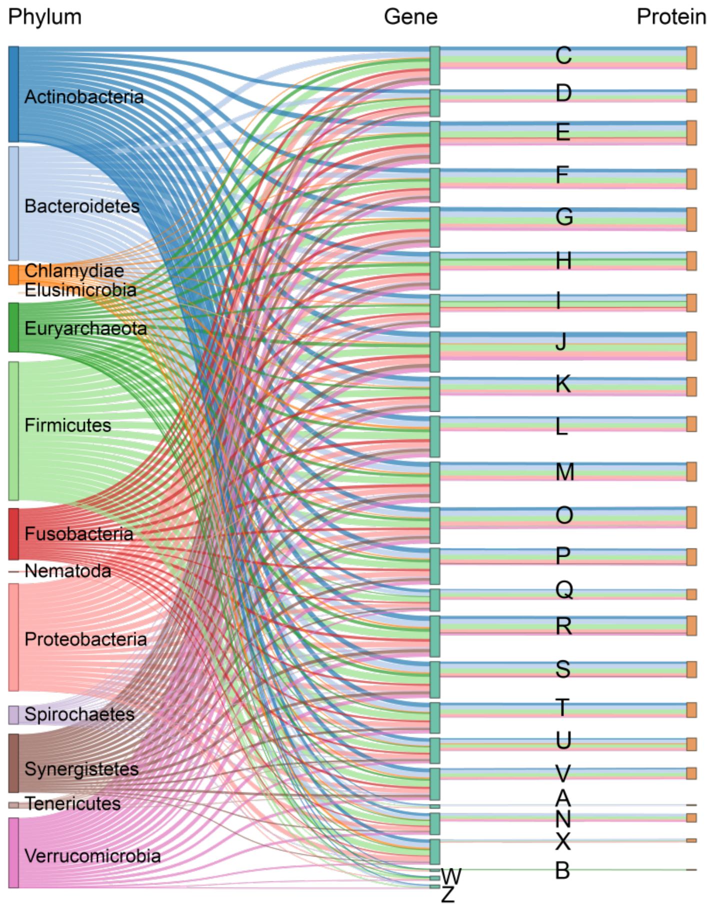

Figure S6. Tripartite plot showing taxonomic and functional relationships between GCN and PCN for individual microbiome sample HM466.

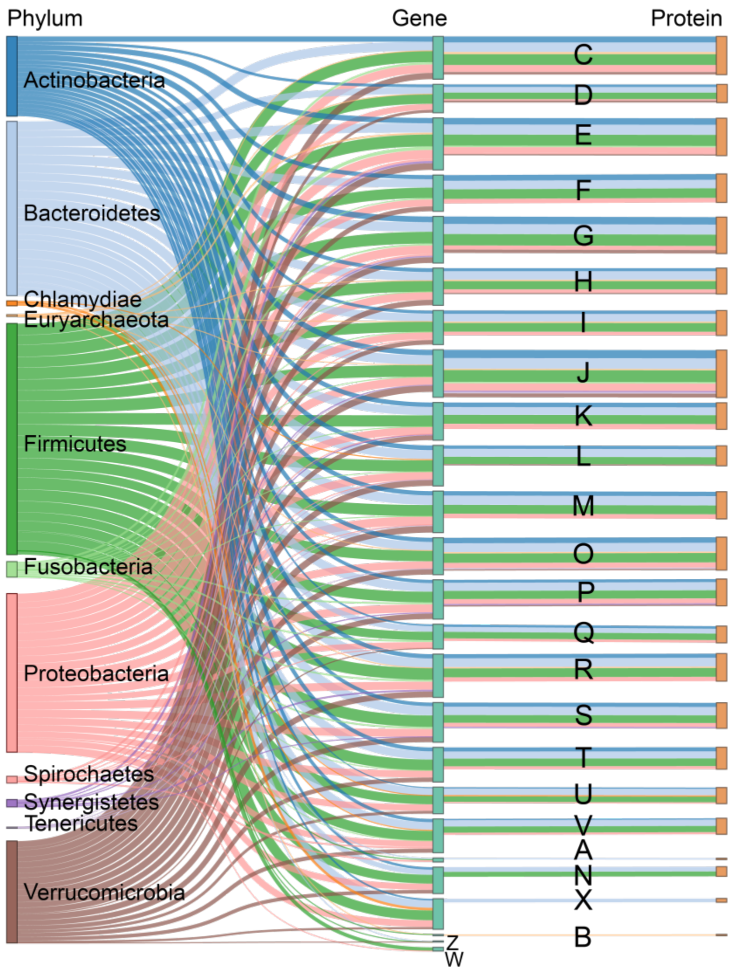

Figure S7. Tripartite plot showing taxonomic and functional relationships between GCN and PCN for individual microbiome sample HM503.

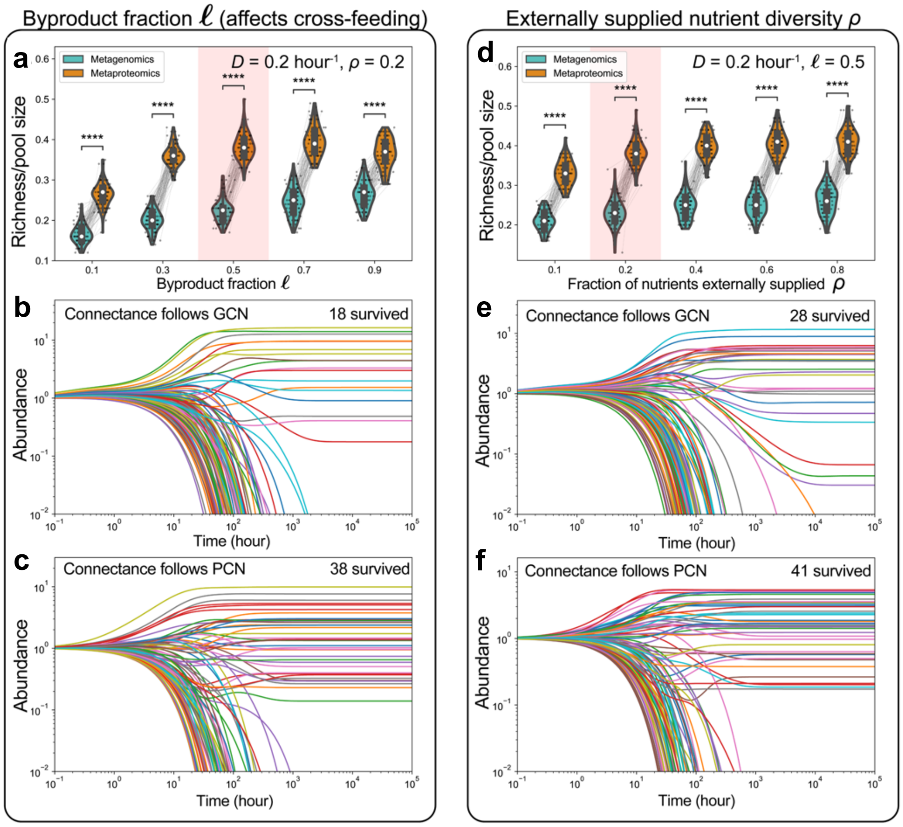

Figure S8. More simulations performed by altering byproduct fraction and externally supplied nutrient diversity.

In addition to **Figure 2e-g** of the main text, more simulations were performed. Byproduct fraction l (**a**), and fraction of externally supplied nutrients ρ (**d**) were simulated with consumption matrices with the same connectance as the GCN (C^{GCN}) or PCN (C^{PCN}). C^{PCN} is generated via a subsampling of the C^{GCN}. The one pair of simulations (for GCN and PCN) in (**a**) and (**d**) are shown as (**b**)-(**c**) and (**e**)-(**f**) respectively. The default set of values is dilution rate D = $0.2\ hour^{-1}$, byproduct fraction l = 0.5, and when 20 out of 100 nutrients are externally supplied ($\rho = 0.2$). Parameters for all panels are as follows: (**a**) D = $0.2\ hour^{-1}$ and ρ = 0.2; (**d**) D = $0.2\ hour^{-1}$ and l = 0.5; (**b**)-(**c**), and (**e**)-(**f**) D = $0.2\ hour^{-1}$, l = 0.5, and ρ = 0.2. Scattered dots and lines linking pairs of dots in (**a**) and (**d**) indicate each simulation paired between $C^{\mathrm{GCN}}$and C^{PCN}. Middle white dot in the box plot denotes median, the lower and upper hinges correspond to the first and third quartiles, the black line ranges from the 1.5 × (interquartile range) below the lower hinge to 1.5 × IQR above the upper hinge, and whiskers represent the maximum and minimum, excluding outliers. Statistical analyses were performed using the two-sided Mann-Whitney-Wilcoxon U Test with Bonferroni correction between genomic capability (GCN) and protein functions (PCN). **** indicate statistical significance at the p < 0.0001 level. N numbers for (**a**) and (**d**): N = 100 times of independent simulations. p values, (**a**): left to right, $p = 1.4\times10^{-33}$, $p = 1.8\times10^{-34}$, $p = 1.9\times10^{-34}$, $p = 2.6\times10^{-34}$, $p = 7.9\times10^{-32}$, (**d**): left to right, $p = 2.0\times10^{-34}$, $p = 2.7\times10^{-34}$, $p = 2.8\times10^{-34}$, $p = 2.1\times10^{-34}$, $p = 2.5\times10^{-34}$. Source data are provided as a Source Data file.

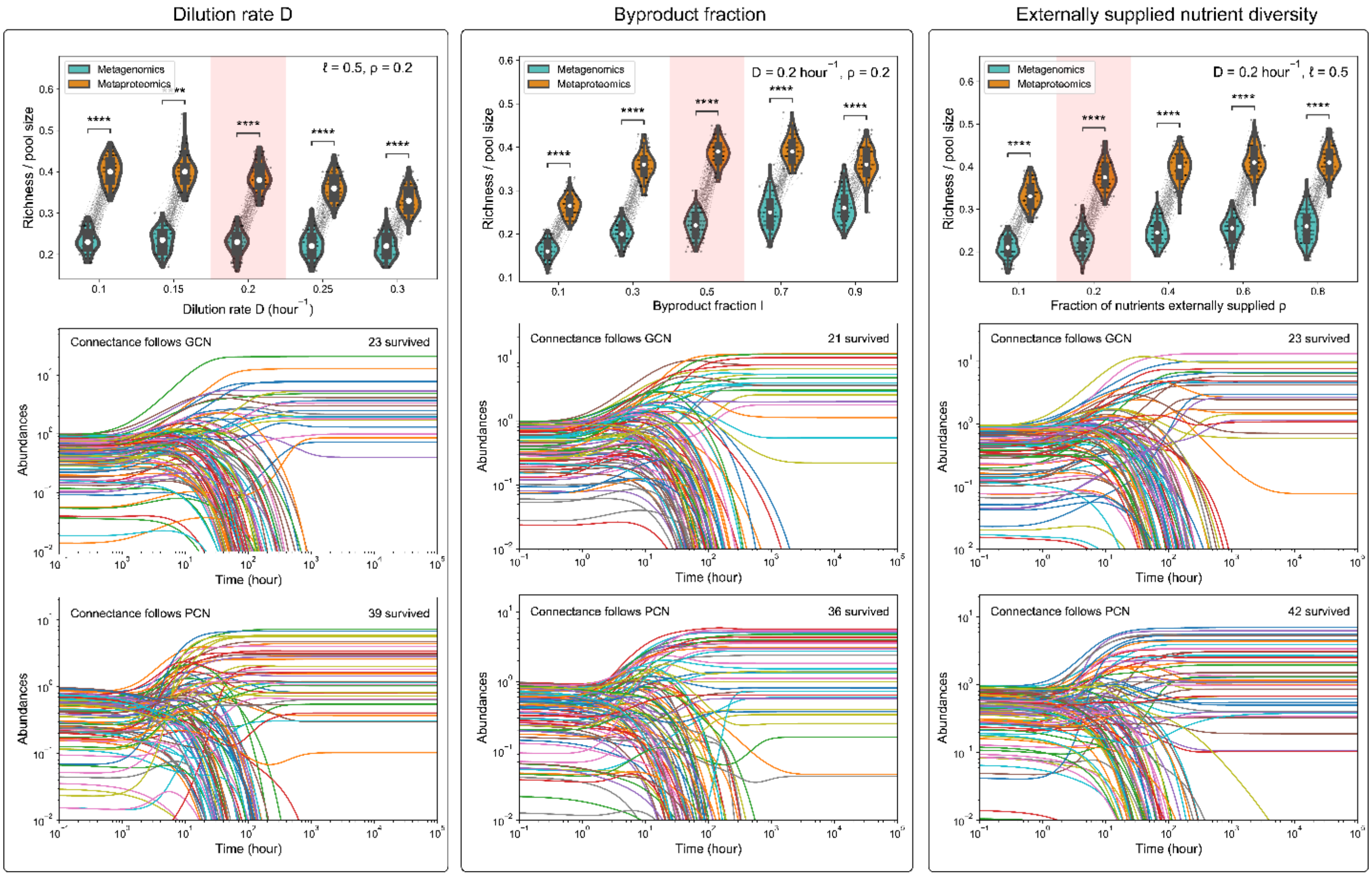

Figure S9. More simulations performed by altering S:M ratio

Simulations performed with different S:M ratios (i.e. the ratio between initial species abundances and initial metabolite/resource concentrations) via drawing initial microbial abundances and resource concentrations from the uniform distribution from 0 to 1. Middle white dot in the box plot denotes median, the lower and upper hinges correspond to the first and third quartiles, the black line ranges from the 1.5 × (interquartile range) below the lower hinge to 1.5 × IQR above the upper hinge, and whiskers represent the maximum and minimum, excluding outliers. Statistical analyses were performed using the two-sided Mann-Whitney-Wilcoxon U Test with Bonferroni correction between genomic capability (GCN) and protein functions (PCN). N = 100 times of independent simulations. *p* values, (**left column**): left to right, $p = 2.0\times10^{-34}$, $p = 2.0\times10^{-34}$, $p = 1.9\times10^{-34}$, $p = 2.4\times10^{-34}$, $p = 6.5\times10^{-34}$, (**middle column**): left to right, $p = 1.7\times10^{-34}$, $p = 1.9\times10^{-34}$, $p = 2.0\times10^{-34}$, $p = 3.0\times10^{-34}$, $p = 7.7\times10^{-31}$, (**right column**): left to right, $p = 1.9\times10^{-34}$, $p = 2.1\times10^{-34}$, $p = 2.9\times10^{-34}$, $p = 2.1\times10^{-34}$, $p = 2.9\times10^{-31}$. Source data are provided as a Source Data file.

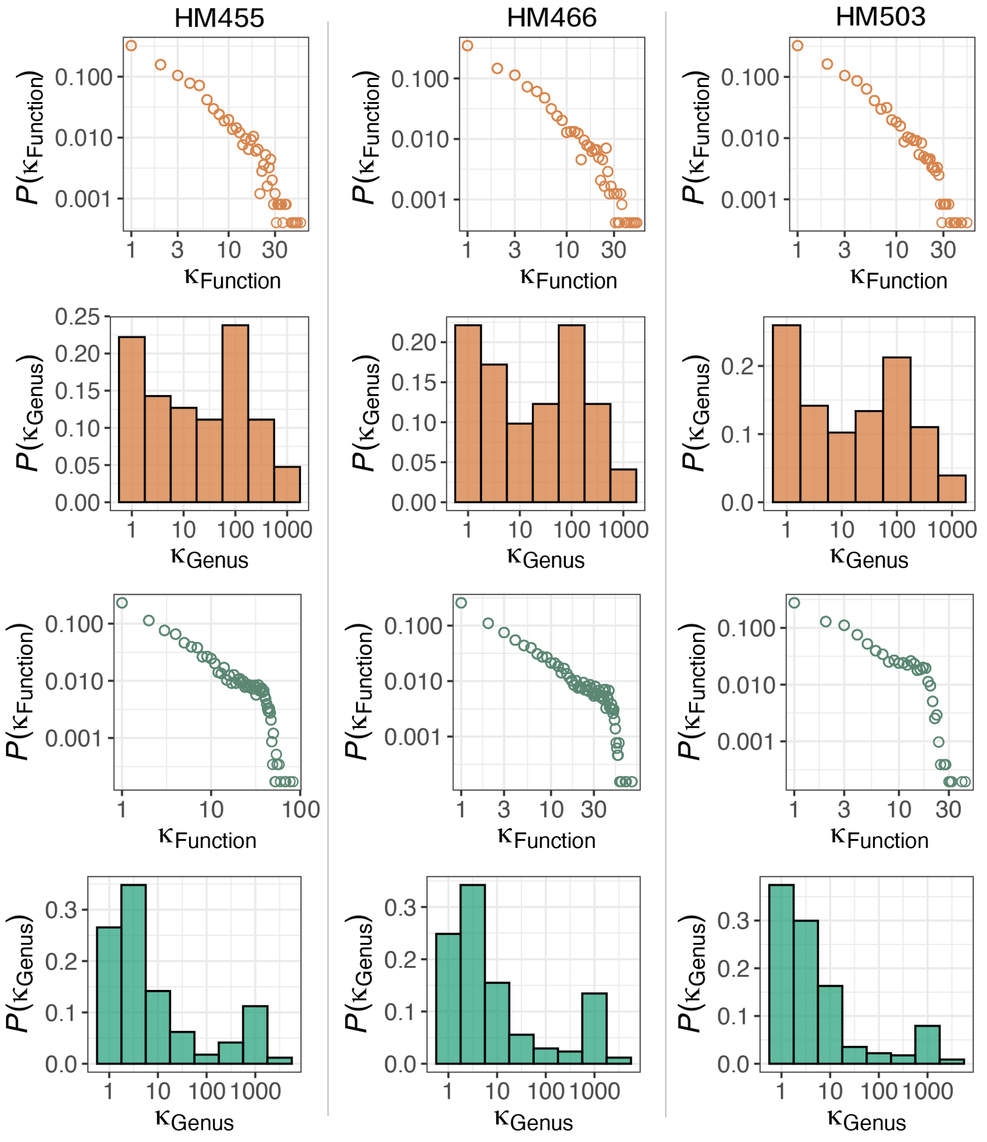

Figure S10. Degree distributions of PCNs and GCNs in the other three microbiomes

The unweighted degree distribution of functions in PCNs (first row), that of genera in PCNs (second row), that of functions in GCNs (third row), and that of genera in GCNs (fourth row) in the individual microbiomes HM455, HM466, HM503.

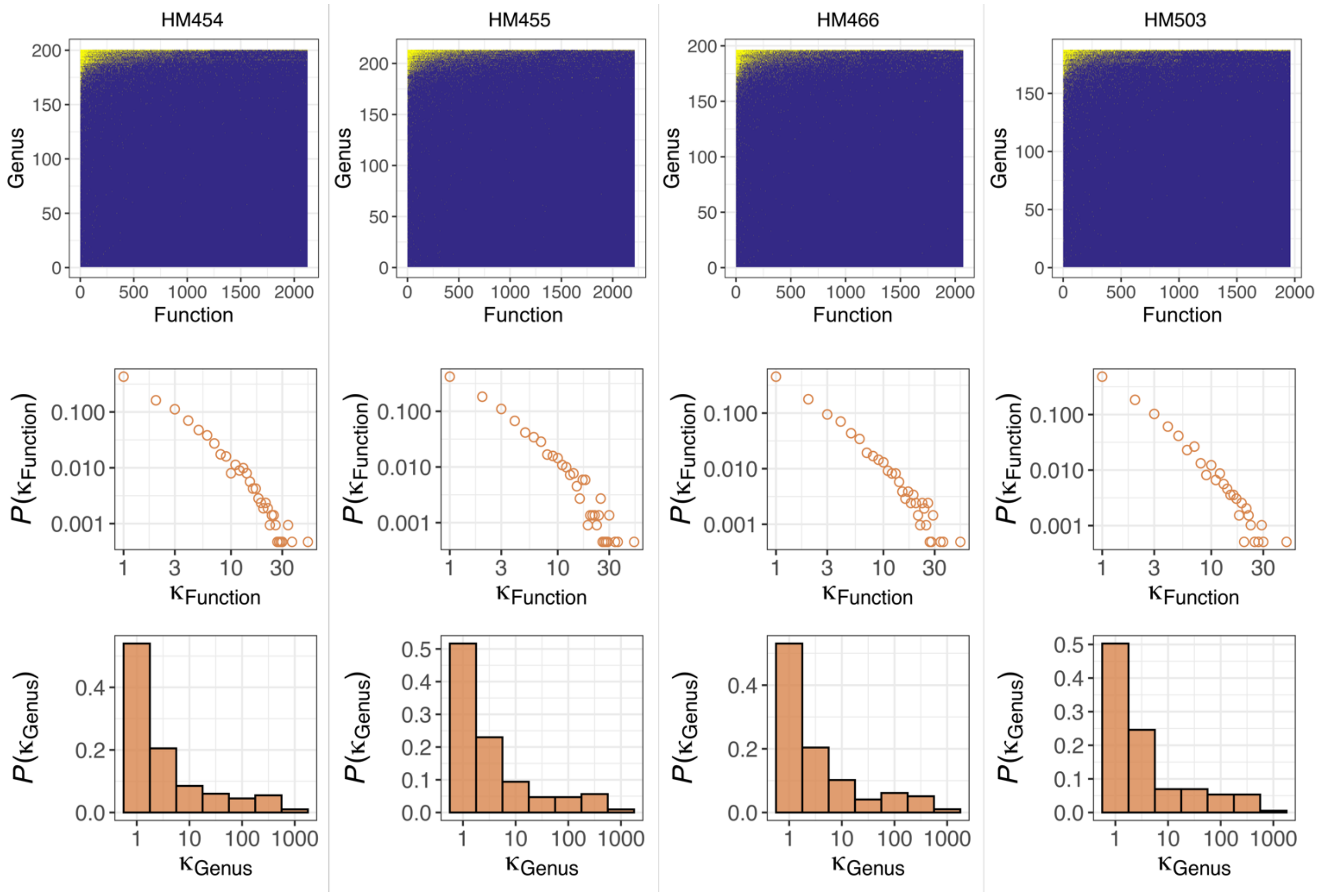

Figure S11. Degree distributions of the four PCNs generated with IGC-based search

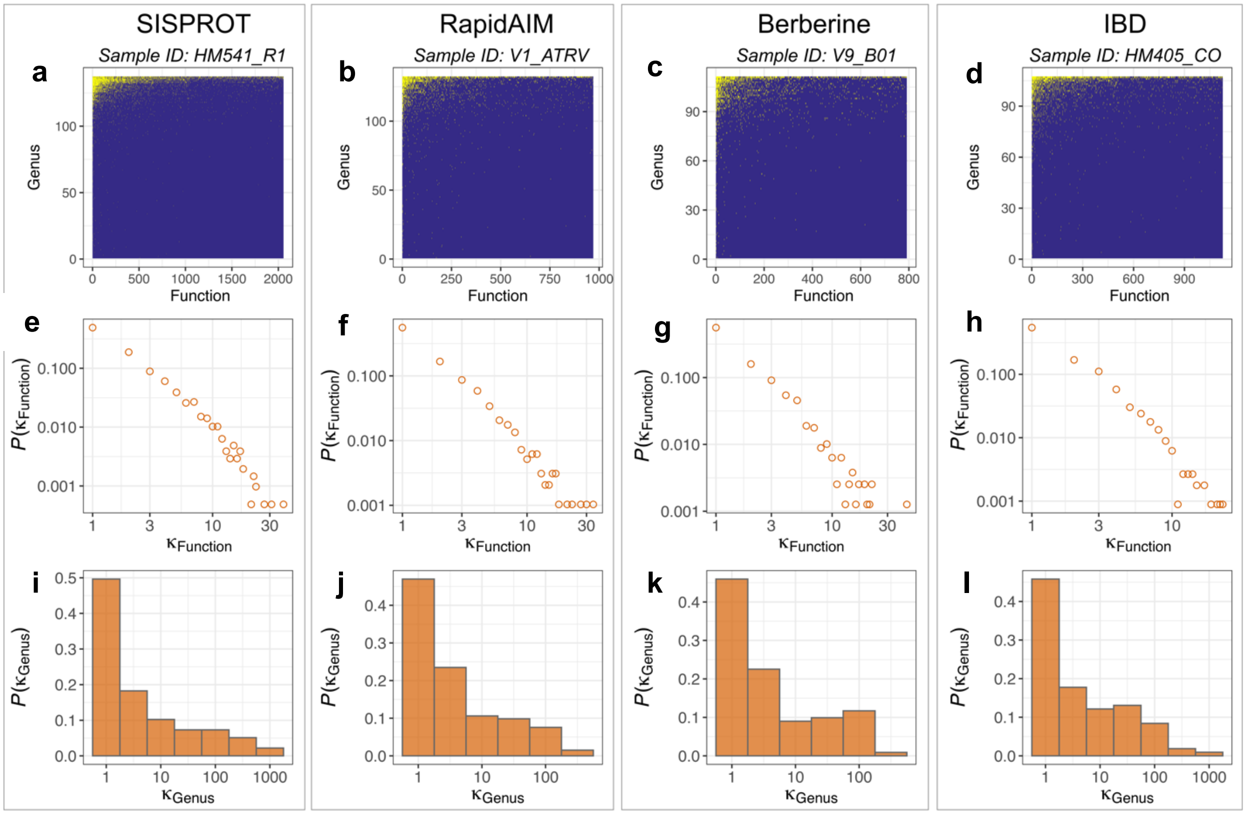

Figure S12. **PCNs and corresponding degree distributions in different metaproteomics datasets**

a-d. Taxon-function incidence matrix of the PCN corresponding to each metaproteomics dataset. The presences of genus-function connections are shown as yellow dots. **e-h**. Unweighted degree distribution of functions corresponding to each metaproteomics dataset. **i-l**. Unweighted degree distribution of genera corresponding to each metaproteomics dataset. Each vertical panel (gray-line box) represents the PCN of the first sample (by alphabet order) in each dataset. We also visualized the incidence matrices and degree distributions of all samples here: https://shiny2.imetalab.ca/shiny/rstudio/PCN_visualizer/

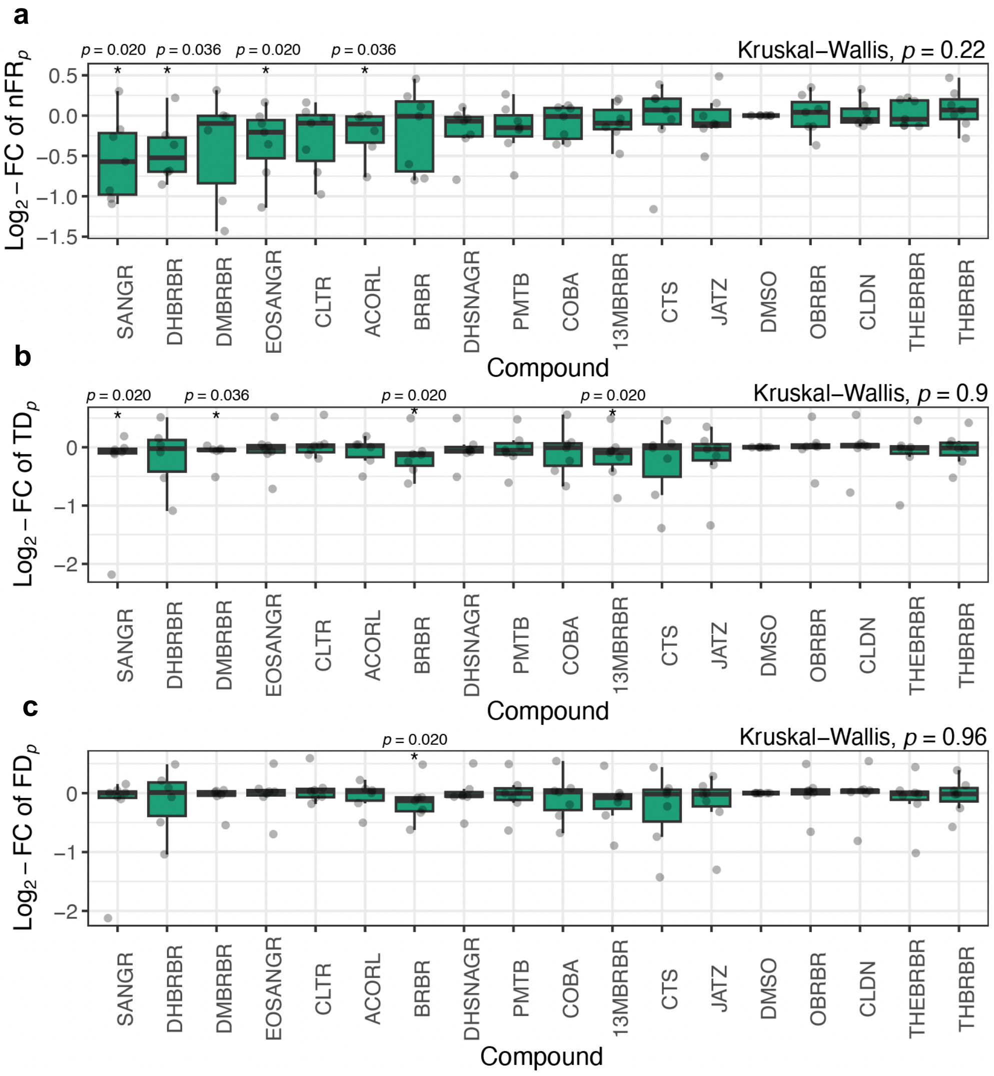

Figure S13. Functional redundancy, taxonomic and functional diversity comparisons in the berberine dataset.

a. Log2-fold change of nFR_p values in comparison to DMSO control samples of each individual. **b**. Log2-fold change of TD_α values in comparison to DMSO control samples of each individual. **c**. Log2-fold change of FD_α values in comparison to DMSO control samples of each individual. N = 7 independent microbiomes (exceptions $N^{(DMBRBR)} = N^{(DHBRBR)} = N^{(ACOL)} = 6$) per compound. Significance of differences between-groups were examined by two-sided Wilcoxon rank-sum test, * and ** indicate statistical significance at the FDR-adjusted *p* < 0.05 and 0.01 levels, respectively. In the box plots, each individual point represents a metaproteomic sample; lower and upper hinges correspond to the first and third quartiles, thick line in the box corresponds to the median, and whiskers represent the maximum and minimum, excluding outliers.

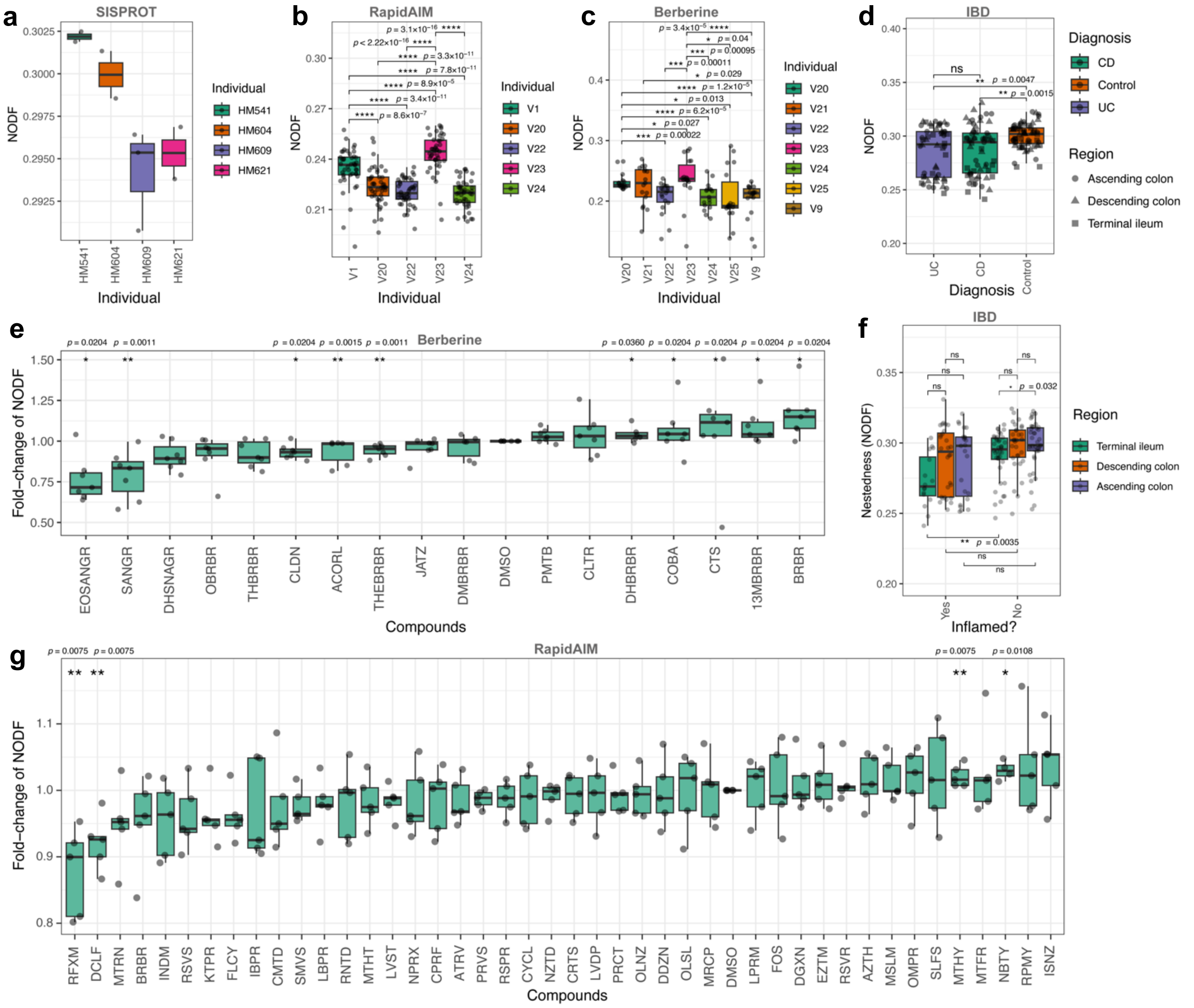

Figure S14. Nestedness metric based on Overlap and Decreasing Fill (NODF) of the metaproteomics datasets.

a. NODF values by individual microbiomes in the SISPROT dataset. **b**. NODF values by individual microbiomes in the RapidAIM dataset. **c**. NODF values by individual microbiomes in the Berberine dataset, N numbers: $N^{(V20)} = N^{(V21)} = N^{(V24)} = 17$, $N^{(V9)} = N^{(V22)} = N^{(V23)} = N^{(V25)} = 18$ compound treated or control microbiomes. **d**. NODF values by diagnosis in the IBD dataset. **e**. NODF fold-change of compound-treated microbiomes in the Berberine dataset, N numbers: $N^{(UC)} = 52$, $N^{(CD)} = 61$, $N^{(Control)} = 63$ samples. **f**. NODF values by inflammation and gut region in the IBD dataset, N numbers: $N^{(Ascending\ colon,\ inflamed)} = 23$, $N^{(Descending\ colon,\ inflamed)} = 28$, $N^{(Terminal\ ileum,\ inflamed)} = 16$, $N^{(Ascending\ colon,\ non\text{-}inflamed)} = 39$, $N^{(Descending\ colon,\ non\text{-}inflamed)} = 30$, $N^{(Terminal\ ileum,\ non\text{-}inflamed)} = 40$ independent metaproteomic analyses. **g**. NODF fold-change of compound-treated microbiomes in the RapidAIM dataset, N = 5 (with exception $N^{(NBTY)} = 4$) biologically

independent microbiomes. *, **, *** and **** indicate statistical significance at the $p < 0.05$, 0.01, 0.001 and 0.0001 levels, respectively, by two-sided Wilcoxon rank-sum test. In the box plots, each individual point represents a metaproteomic sample; lower and upper hinges correspond to the first and third quartiles, thick line in the box corresponds to the median, and whiskers represent the maximum and minimum (excluding outliers). Source data are provided as a Source Data file.

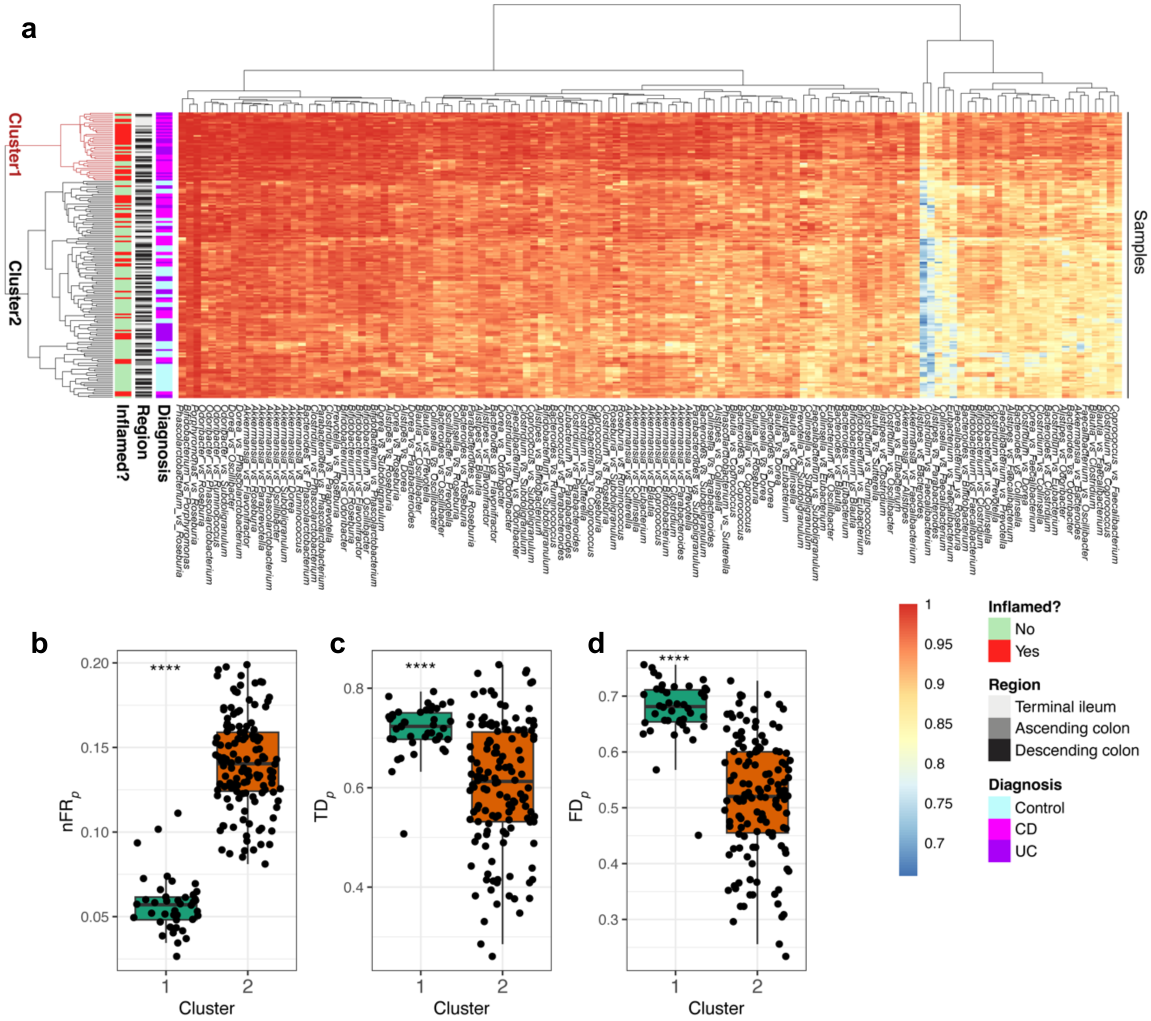

Figure S15. Comparison of between-genera dij values across all IBD samples.

a. Heatmap showing d_{ij} values between genera across samples in the IBD dataset. **b-d**. nFR_p, TD and FD values between cluster 1 and cluster 2. N numbers, $N^{(cluster\ 1)} = 42$, $N^{(cluster\ 2)} = 134$. Lower and upper hinges correspond to the first and third quartiles, thick line in the box corresponds to the median, and whiskers represent the maximum and minimum, excluding outliers. Wilcoxon rank sum test (two-sided) was performed, **** indicate statistical significance at the p < 0.0001 level. *p* values, (**b**): $p = 2.0\times10^{-16}$, (**c**): $p = 1.8\times10^{-8}$, (**d**): $p = 3.1\times10^{-16}$. Source data are provided as a Source Data file.

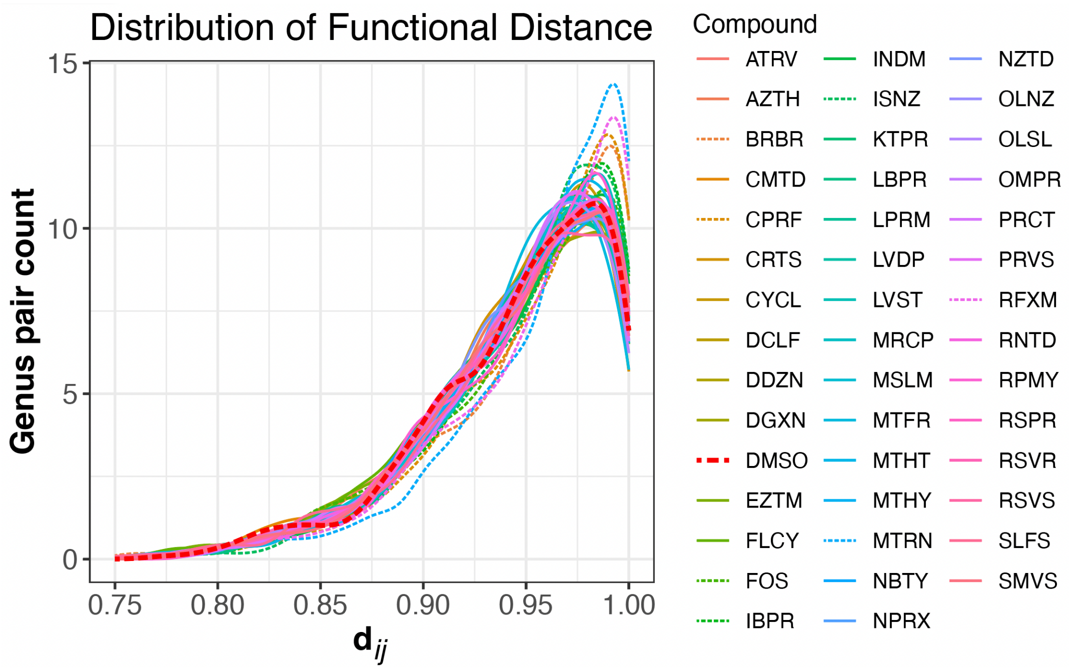

Figure S16. Distribution of d_{ij} values by compounds in the RapidAIM dataset.

Each distribution line was plotted using the mean value across individual microbiomes (N=5) corresponding to the control (DMSO, red dashed line) or other compounds. Compounds shown in dashed lines, i.e. berberine (BRBR), ciprofloxacin (CPRF), fructo-oligosaccharide (FOS), ibuprofen (IBPR), isoniazid (ISNZ), metronidazole (MTRN) and rifaximin (RFXM) showed overall shifts in the distribution.

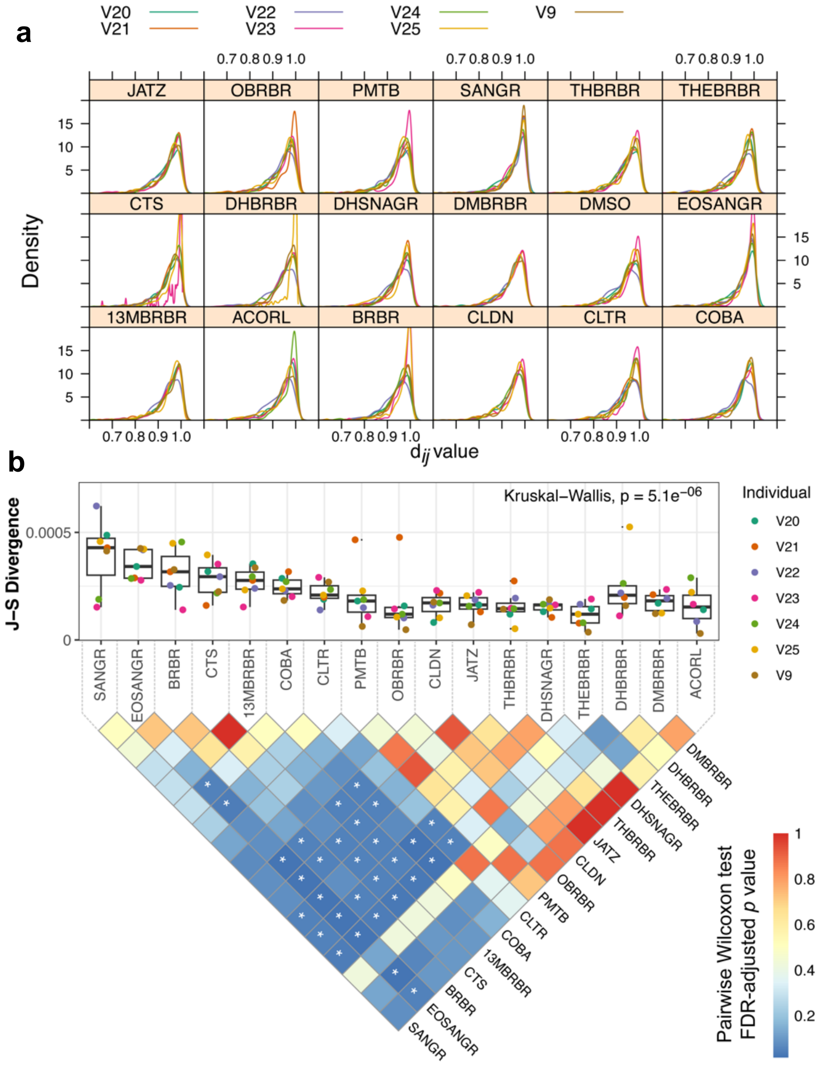

Figure S17. Between-genera functional distances in the Berberine dataset.

a. d_{ij} distribution by different berberine analogues and by different individual microbiomes. **b**. J-S divergence between the d_{ij} distribution in the control (DMSO) and that of the other compounds. Lower and upper hinges in the boxplots correspond to the first and third quartiles, thick line in the box corresponds to the median, and whiskers represent the maximum and minimum (excluding outliers). Kruskal-Wallis test result indicated that overall the compounds had heterogeneous levels of J-S divergence with the DMSO. Between-compound comparisons of the J-S divergence values were performed by a Pairwise Wilcoxon Rank Sum Tests, * indicates statistical significance at the FDR-adjusted $p < 0.05$ level. N = 7 independent microbiomes (exceptions $N^{(DMBRBR)} = N^{(DHBRBR)} = N^{(ACOL)} = 6$) per compound. The results were based on microbial genera of the top 95% overall protein biomass in the dataset. Source data are provided as a Source Data file.

Supplementary Note 1

Generating PCN from MaxQuant and MetaLab search results

Through the Metapro-IQ workflow, we obtained the ProteinGroups.txt, and peptides.txt tables. The Protein groups table (generated by MaxQuant) contains information on the identified proteins, and identifiers of peptide sequence associated to each protein group. Through MetaLab, we further obtained MetaLab_peptide.xlsx table and function.csv tables. These tables are inter-connected through peptide sequences, peptide id numbers, and protein IDs. Therefore, we were able to match taxon and function by combining these result tables.

Step-by-step workflow: A detailed step-by-step workflow is described below, as well as illustrated in **Supplementary Note Figures N1** and **N2**.
Step 1. The MetaLab_peptide.xlsx table (or set of tables) contain peptide sequences and the taxonomic matching according to the MetaLab pep2taxon database. And the peptide.txt file includes a column of unique peptide IDs for each peptide sequence. These two tables were first combined to generate a peptide_ID_to_taxon table.
Step 2. Each protein group in the ProteinGroup.txt table correspond to a series of peptide IDs, we are therefore able to link each protein group to the taxonomic information by querying these peptide IDs from the peptide_ID_to_taxon table. Protein group intensities were also kept in this table.
Step 3. The genus level information was summarized for each protein group to generate a ProteinGroup_genus_intensity table. Here, we approximately consider that the peptides corresponding to each protein group are derived from a same genus. We validated that this approach has a confidence of 98.4% at the genus level based on the ultra-deep metaproteomics dataset (**Supplementary Note Table N1**).
Step 4. Next, annotated functions were taken from the top 1 protein in each protein group (function_top1 table). We validated that functions of proteins in each protein group have an agreement of 97.7% based on the ultra-deep metaproteomics dataset. In addition, top 1 protein in each protein group is considered the most confident protein identification, given by its number of identified peptides and E values.
Step 5. The function_top1 table was combined with the protein ProteinGroup_genus_intensity table to generate a ProteinGroup_function_taxon_intensity table.
Step 6. The ProteinGroup_function_taxon_intensity table can then be converted into PCN in the form of a bipartite network or an incidence matrix $\mathbf{P} = [\mathrm{P_{ia}}]$.

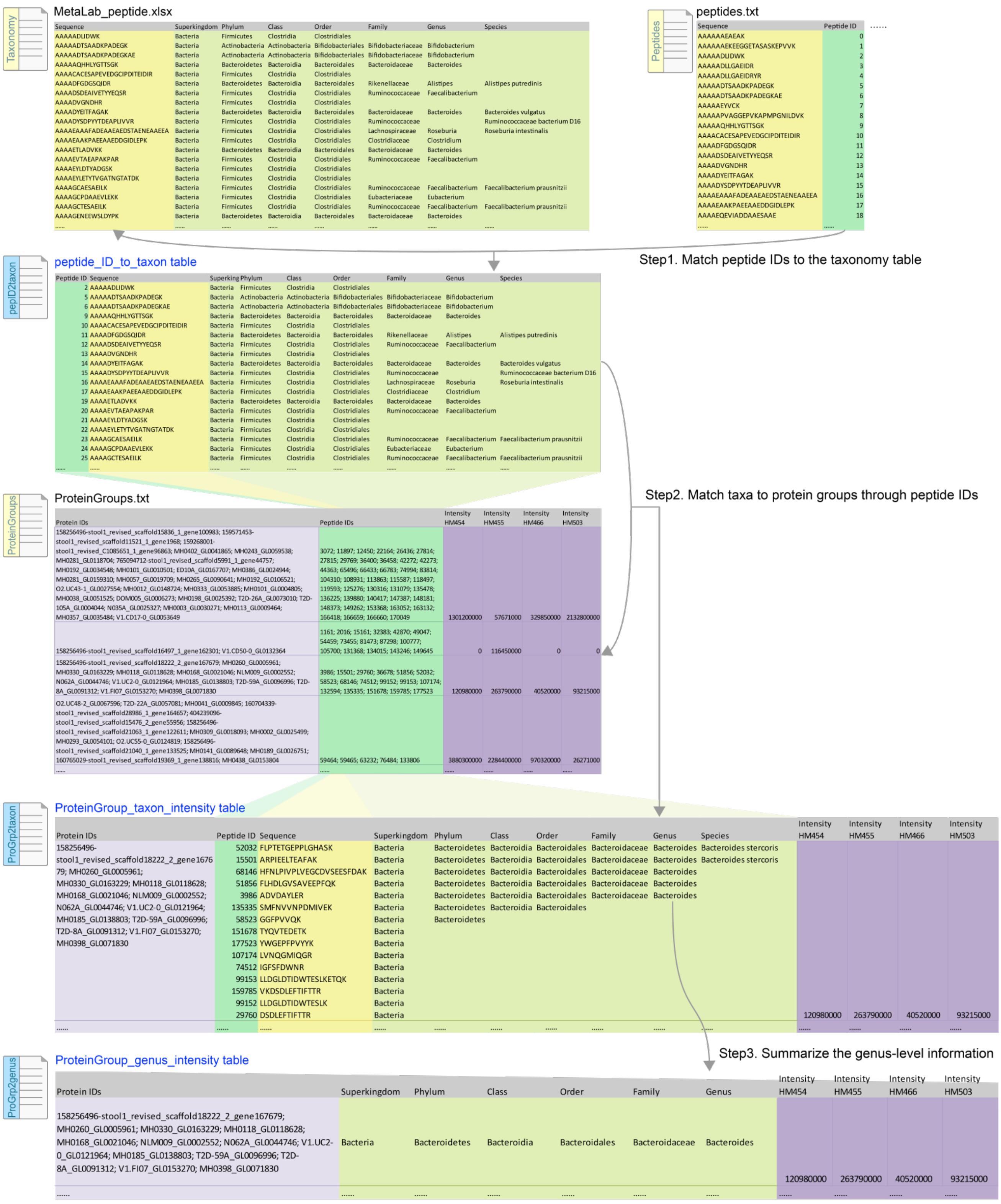

(Yellow documents represent database search outputs, and blue documents represent tables generated during the matching process.)

Figure N1. Step-by-step workflow for PCN generation, part I.

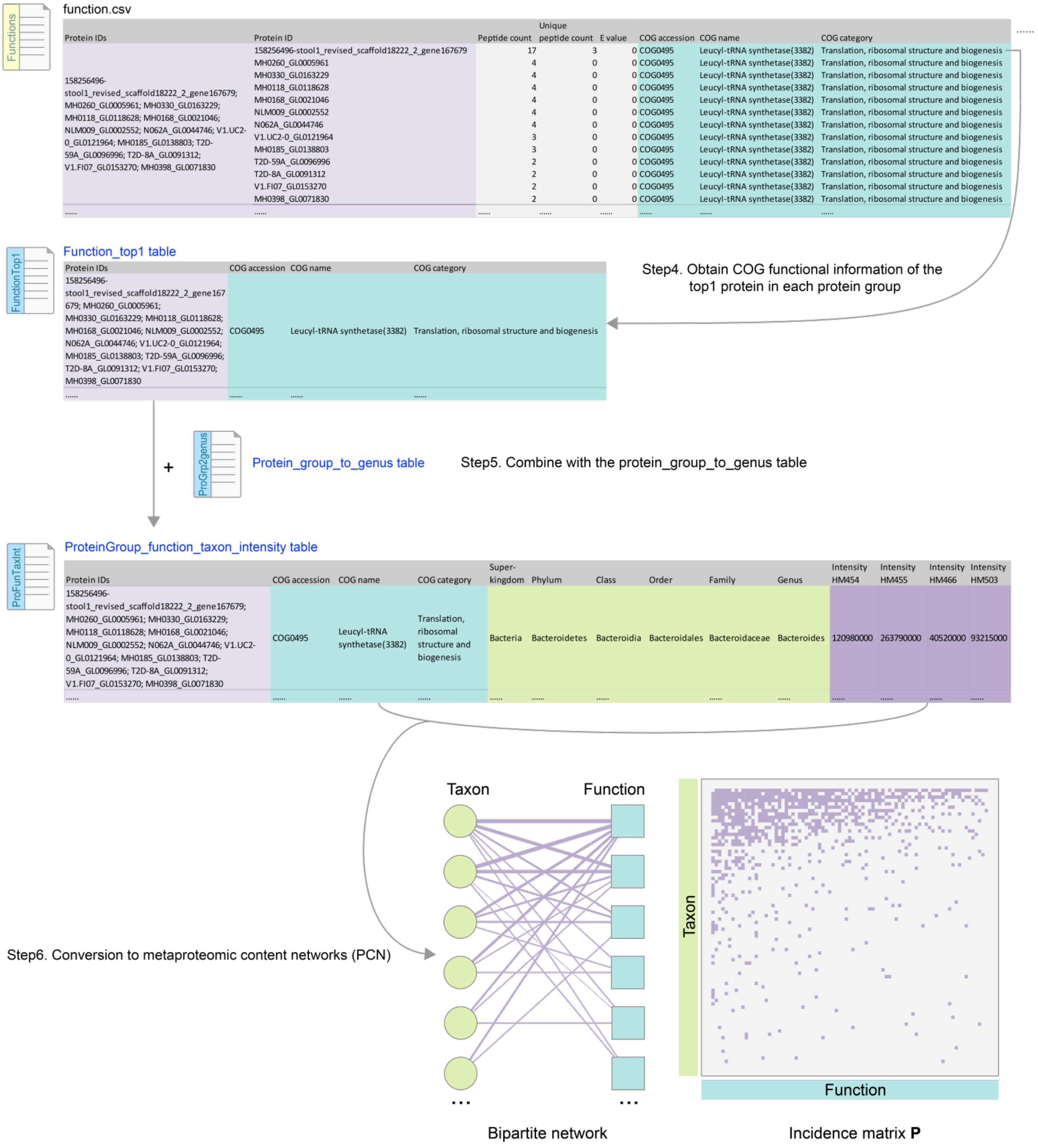

Figure N2. Step-by-step workflow for PCN generation, part II.

Table N1. Confidence of protein group-to-taxon matching

Taxonomic level:	Super-kingdom	Phylum	Class	Order	Family	Genus	Species
**All unique pairs of matches at this level**	46,592	44,894	41,269	41,016	29,425	26,748	15,477
**Protein groups matched to only one taxon at this level**	46,553	44,491	40,900	40,778	28,855	26,322	15,104
**ProteinGroup% matched to a unique taxon at this level**	99.9%	99.1%	99.1%	99.4%	98.1%	98.4%	97.6%
**ProteinGroup% matched to more than one taxa at this level**	0.1%	0.9%	0.9%	0.6%	1.9%	1.6%	2.4%

Note: Numbers of matches were calculated using the ultra-deep metaproteomics dataset.

Supplementary Note 2

To determine the best method for functional annotations in the PCNs, we looked into the use of four different functional annotations (COG, KEGG, MetaCyc and CAZyme) of proteins identified based on the IGC database and the MetaPro-IQ approach. The use of comprehensive functional databases COG and KEGG yielded similar results (**Supplementary Note Figure N3A-B)**. In particular, those different workflows yield highly similar network topologies and functional redundancy comparisons between GCN and PCN. MetaCyc is also a powerful functional annotation tool metaproteomic pathway analysis. However, we emphasize that it is not applicable in this current study. The reason is simple: in MetaCyc the list of functions annotated to a protein ID can be a list of various synonyms without a unified identifier to be used for summarization for generating the PCN (**Supplementary Note Figure N4**). We finally looked into CAZymes as a representative of enzymes within specific functional classes. Interestingly, despite that CAZyme-PCNs contain a much smaller proportion of proteins (~5%) compared to the full PCN, we still observed a highly nested network topology. More interestingly, the functional redundancy values are close to the values calculated from COG or KEGG (**Supplementary Note Figure N3C**).

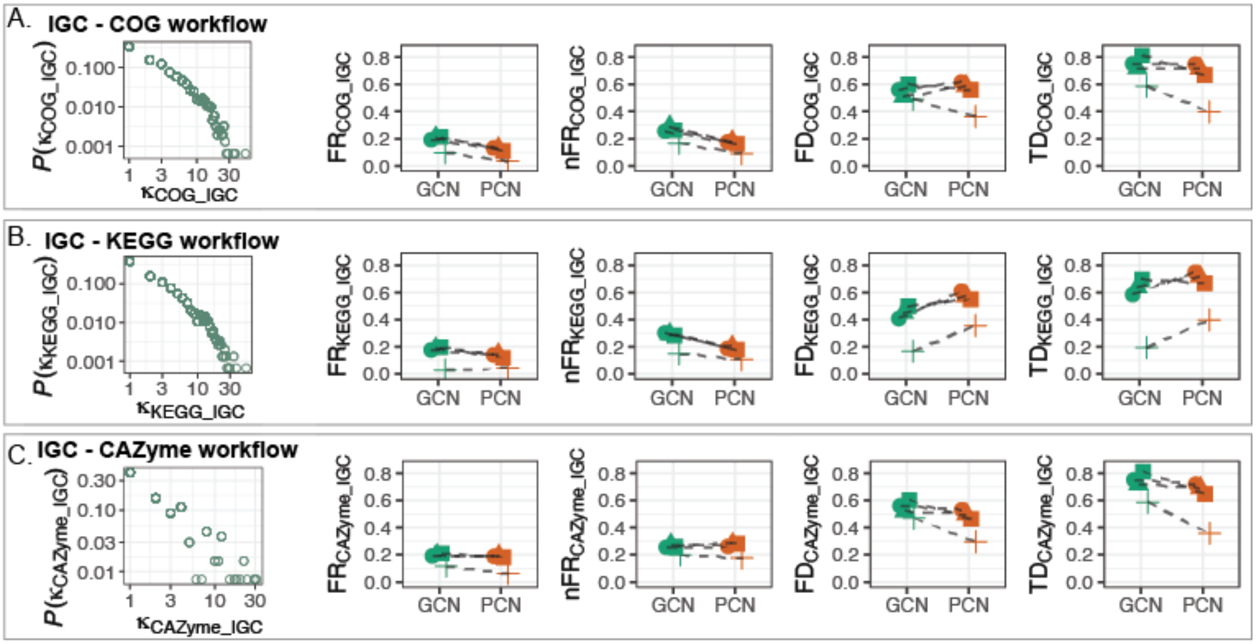

Figure N3. Comparison of network topology and FR_p, nFR_p, FD_p, TD_p computed using different functioal annotation databases.

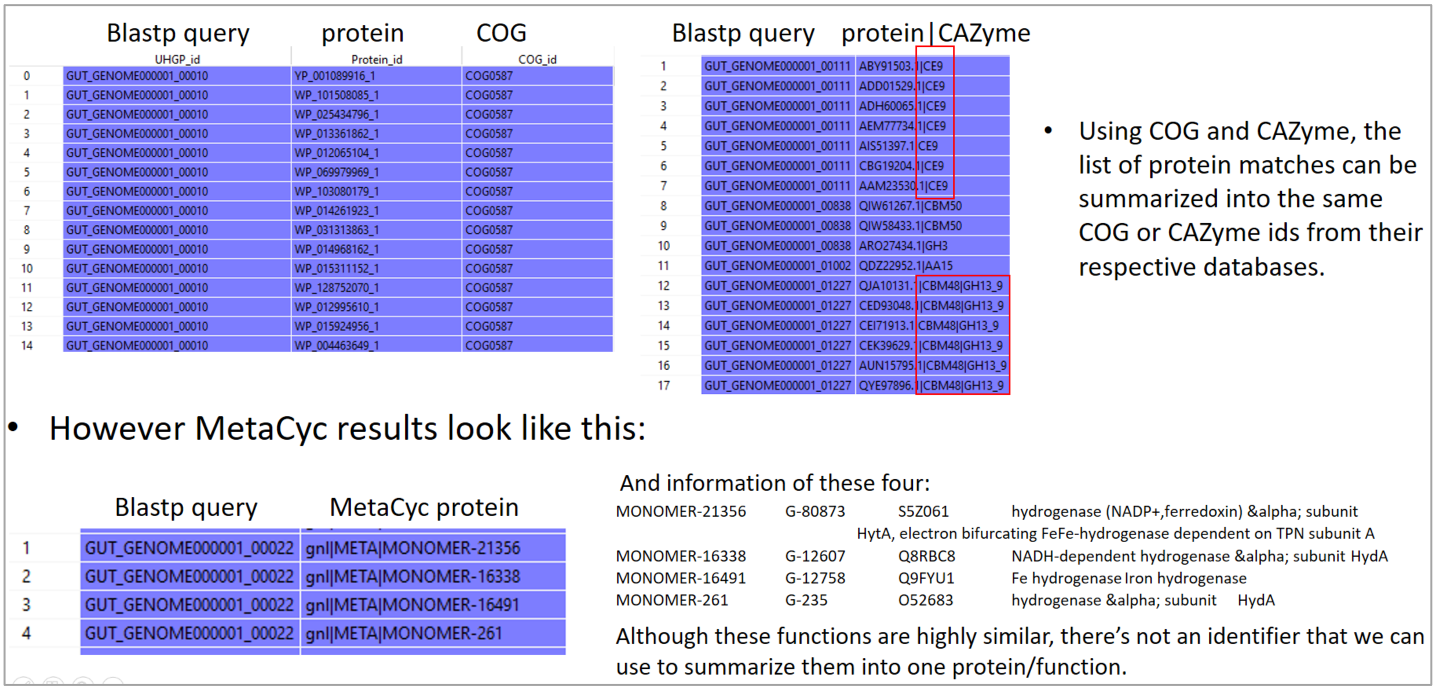

Figure N4. Examples and explanations showing why COG/KEGG_KO/CAZyme can be used for GCN/PCN computation, but the use of MetaCyc is impossible here.

We also examined the performance of these different functional annotations (COG, KEGG, and CAZyme) in quantifying FR_p differences in the RapidAIM dataset (**Supplementary Note Figure N5**). We found that both the COG and KEGG workflows showed significant decreased in nFR_p (drug rifaximin (RFXM) vs dimethyl sulfoxide (DMSO) as an example). CAZyme failed to detect the differences. This might be due to the fact that comprehensive databases COG and KEGG help better capture the whole community-level functional redundancy profile.

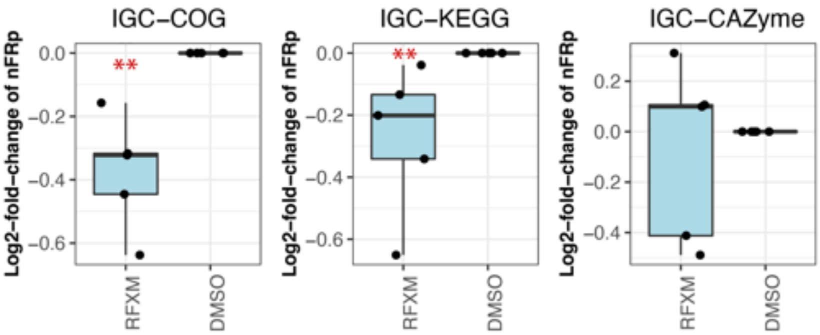

Figure N5. Comparison of nFR_p changes determined by different workflows in the RapidAIM dataset. Comparison between RFXM (rifaximin) treatment and DMSO (control) groups are shown. N = 5 independent microbiomes per group. Lower and upper hinges correspond to the first and third quartiles, thick line in the box corresponds to the median, and whiskers represent

the maximum and minimum. ** indicate statistical significance at the $p < 0.01$ level by two-sided Wilcoxon rank sum test. p values: IGC-COG, $p = 0.0075$; IGC-KEGG, $p = 0.0075$.

We next studied the response of functional distance d_{ij} values using the different functional annotations. We performed the same analysis as in **Figure 5g** of the main text (i.e. IGC-COG workflow) using the IGC-KEGG and IGC-CAZyme workflows. J-S divergence of functional distances between taxa did not show a significant difference among the drugs in IGC-KEGG and IGC-CAZyme results (**Supplementary Note Figure N6A-C**). Therefore, results suggest that KEGG and CAZyme are not as sensitive as COG in comparing differences in microbiome functional networks.

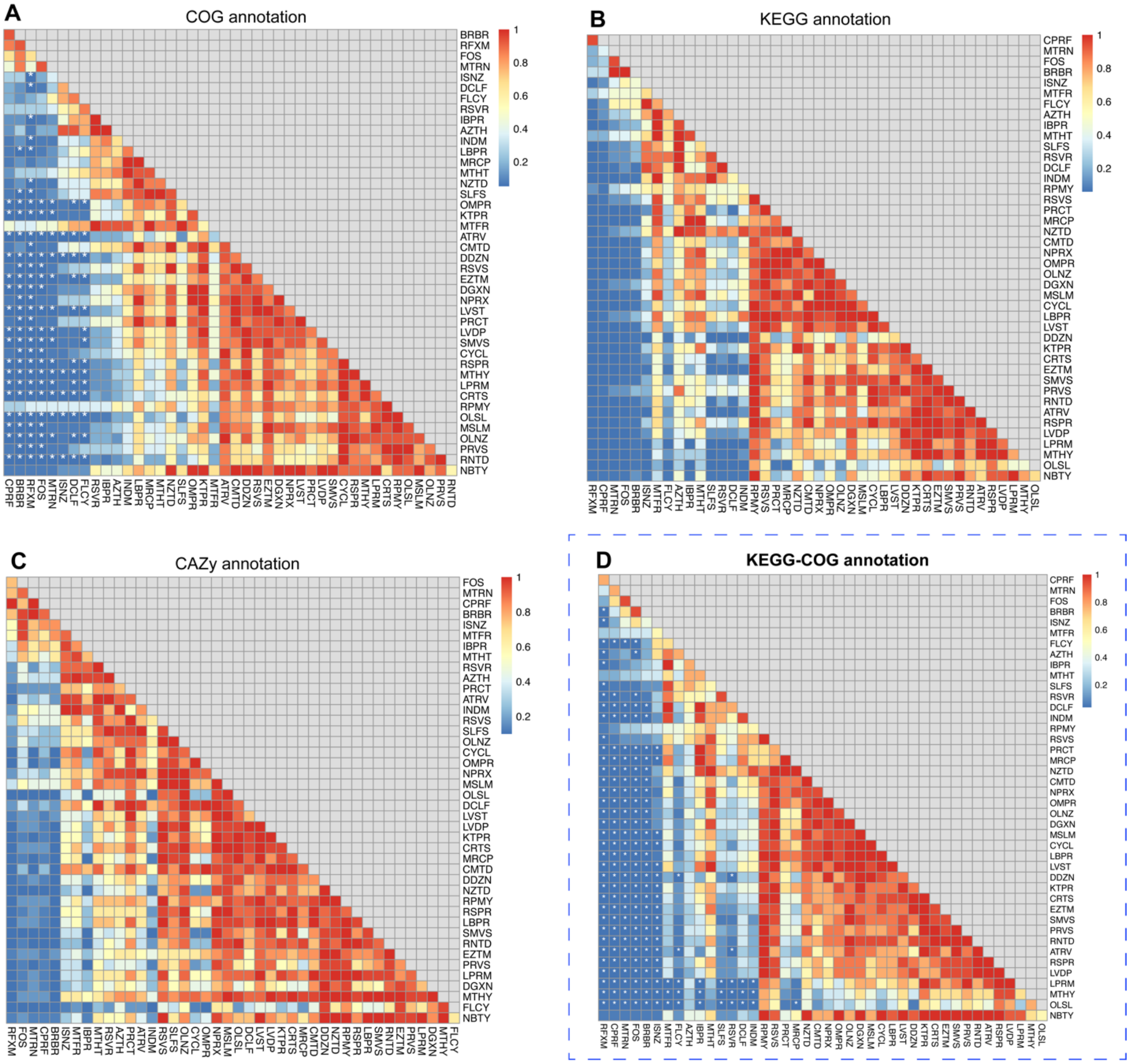

Figure N6. Comparison of different functional annotation methods in sensitivity of detecting functional distances variations. A. COG annotation showed significant alteration of

between-genera d_{ij} distributions in response to drug treatments (J-S divergence). **B-C.** Using KEGG and CAZymes, despite the observation of similar patterns in the p-value heatmap, we did not observe any significant difference in J-S divergence between drugs (no asterisks shown). **D**. The KEGG-COG annotation method showed significant alteration of between-genera d_{ij} distributions in response to drug treatments, in agreement with panel (A), COG-based annotations. Asterisks indicate statistical significance at the 0.05 level (FDR adjusted *p* value, Pairwise Wilcoxon test).

However, notably, the COG database has a lower functional granularity compared to the KEGG database. Using the ultra-metaproteomic dataset as an example, we found that while 75.9% COGs correspond to unique KOs, the remaining 24.1% COGs were matched to more than one KOs (**Supplementary Note Figure N7**). Although COG compromises functional granularity, the COG provides a higher annotation coverage than the KEGG (for example, for the deep metaproteomics dataset of the four individuals, there were a total of 50,216 protein groups identified. 46,095 (91.7%) of these protein groups were successfully annotated with COGs, while only 37,795 (75.3%) of these protein groups were annotated with KOs). Some functions in COG belong to the categories of 'General function prediction only' and 'Functions unknown' and thus can be included in our FR_p computation. The higher coverage of proteins with the COG database may be the reason that the COG annotation yielded the most sensitive results in FRp comparisons compared to other functional annotation methods.

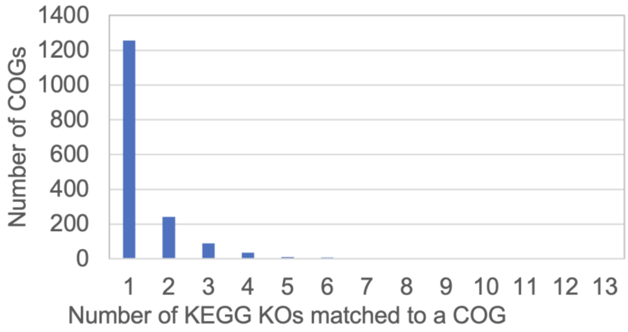

Figure N7. Comparison between KEGG KO and COG matches

Can we combine the merit of high protein coverage of the COG annotation, and the merit of good functional granularity of the KEGG annotation? We found a solution to this question by complementing KEGG annotations with COG annotations. In more details, we first used KEGG to annotate functions to protein groups. Next, for those that could not be annotated with a KO, the annotations were complimented with COG when a protein-COG match presents. We show that results obtained with the COG annotation can be well-reproduced by the KEGG-COG annotation (**Supplementary Note Figure N6D**). Therefore, in this manuscript, we selected the KEGG-COG annotation strategy for the analysis of results.

Supplementary Tables

Table S1. Details of the samples used for ultra-deep metaproteomic analysis

Table S2. RapidAIM dataset compound information

Table S3. Berberine dataset compound information

Table S4. Informations of the four metaproteomics datasets

Table S5. Analysis of nFR variance contributed by region,diagnosis and inflammation in the IBD dataset

Table S6. Analysis of nFR variance contributed by individuals and compounds in the RapidAIM dataset

Table S7. Analysis of nFR variance contributed by individuals and compounds in the Berberine dataset

Table S8. Analysis of d_{ij} variance contributed by region, diagnosis and inflammation in the IBD dataset

Table S9. Analysis of d_{ij} variance contributed by individual and compounds in the RapidAIM dataset

Table S9. Analysis of d_{ij} variance contributed by individual and compounds in the Berberine dataset

Table S1. Details of the samples used for ultra-deep metaproteomic analysis

Subject	Gender	Age (years)	Body weight (kg)	Height (cm)	Colonoscopy Location
HM454	Male	12	56	155.5	Ascending colon
HM455	Female	15	61	156.9	Ascending colon
HM466	Female	17	64.3	160	Ascending colon
HM503	Female	16	63	157.7	Ascending colon

Table S2. RapidAIM dataset compound information

#	Compound	Abbreviation used
1	Metformin hydrochloride	MTFR
2	Berberine chloride	BRBR
3	Rifaximin	RFXM
4	Metronidazole	MTRN
5	Isoniazid	ISNZ
6	Ciprofloxacin	CPRF
7	Resveratrol	RSVR
8	Daidzein	DDZN
9	Risperidone	RSPR
10	Olanzapine	OLNZ
11	Methylprednisolone	MTHY
12	Cortisone	CRTS
13	Olsalazine sodium	OLSL
14	Sulfasalazine	SLFS
15	Mesalamine	MSLM
16	Diclofenac sodium	DCLF
17	Indomethacin	INDM
18	Ibuprofen	IBPR
19	Ketoprofen	KTPR
20	Naproxen sodium	NPRX
21	Ranitidine hydrochloride	RNTD
22	Nizatidine	NZTD
23	Cimetidine	CMTD
24	Lovastatin	LVST
25	Simvastatin	SMVS
26	Pravastatin sodium	PRVS
27	Atorvastatin Calcium	ATRV
28	Rosuvastatin	RSVS
29	Azathioprine	AZTH
30	Mercaptopurine	MRCP
31	Cyclophosphamide monohydrate	CYCL
32	Methotrexate hydrate	MTHT
33	Lubiprostone	LBPR
34	Ezetimibe	EZTM
35	Rapamycin	RPMY
36	Omeprazole	OMPR
37	Paracetamol (Acetaminophen)	PRCT
38	Digoxin	DGXN
39	5-Fluorocytosine	FLCY
40	Loperamide oxide monohydrate	LPRM
41	Levodopa	LVDP
42	Na-butyrate	NBTY
43	FOS (Fructooligosaccharide)	FOS

Table S3. Berberine dataset compound information

#	Compound name	Abbreviation used
1	Tetrahydroepiberberine	THEBBR
2	13-Methylberberine Chloride	13MBBR
3	Demethyleneberberine	DMBBR
4	Oxyberberin	OBBR
5	Tetrahydroberberine	THBBR
6	Dihydroberberine	DHBBR
7	Columbamine	COBA
8	Jatrorrhizine	JATZ
9	Coptisine	CTS
10	Palmatrubine	PMTB
11	Sanguinarine	SANGR
12	Acetylcorynoline	ACORL
13	Chelerythrine	CLTR
14	6-Ethoxysanguinarine	EOSANGR
15	Chelidonine	CLDN
16	Dihydrosanguinarine	DHSNAGR
17	Berberine Chloride	BRBR

Table S4. Informations of the four metaproteomics datasets

Dataset name in brief	Sample type	Bacterial cell lysis and protein extraction	Fractionation method	Mass spectrometer (MS) model	MS run time per sample (minute)	Unique peptides per sample (Mean ± SD)	Protein groups per sample (Mean ± SD)
SISPROT	Fecal sample	Bacterial cells were lysed in urea-Tris-HCl buffer containing 4% SDS, proteins were precipitated and washed before trypsin digestion	SISPROT workflow	Orbitrap Fusion (ThermoFisher Scientific)	1,300	44,922 ± 8,201	20,558 ± 993
RapidAIM	Cultured fecal sample	Bacterial cells were lysed in urea-Tris-HCl buffer, cell lysate were directly used for trypsin digestion	No fractionation	Q Exactive (ThermoFisher Scientific)	90	15,017 ± 3,654	6,684 ± 998
Berberine	Cultured fecal sample	Bacterial cells were lysed in urea-Tris-HCl buffer, cell lysate were directly used for trypsin digestion	No fractionation	LTQ-Orbitrap XL (Thermo Electron)	240	4,345 ± 1,368	5,612 ± 956
IBD	Intestinal aspirate sample	Bacterial cells were lysed in urea-Tris-HCl buffer containing 4% SDS, proteins were precipitated and washed before trypsin digestion	No fractionation	Q Exactive (ThermoFisher Scientific)	240	32,882 ± 8, 836	14,603 ± 3,328

Table S5. Analysis of nFR variance contributed by region,diagnosis and inflammation in the IBD dataset

one-way ANOVA test, aov(nFR ~ region+region:Inflammed+Diagnosis, data = data)

	Df	Sum Sq	Mean Sq	F value	Pr(>F)	Signif.
Region	2	0.00684	0.003422	2.053	0.13153	
Diagnosis	2	0.03215	0.016077	9.648	0.000108	***
Region:Inflammed	3	0.01462	0.004874	2.925	0.035447	*
Residuals	168	0.27996	0.001666			

Signif. codes: 0 ‘***’ 0.001 ‘**’ 0.01 ‘*’ 0.05 ‘.’ 0.1 ‘ ’ 1

Table S6. Analysis of nFR variance contributed by individuals and compounds in the RapidAIM dataset

one-way ANOVA test, aov(nFR ~ Individual + Drug, data = data)

	Df	Sum Sq	Mean Sq	F value	Pr(>F)	Signif.
Individual	4	0.019238	0.00481	85.403	<2.00E-16	***
Drug	43	0.007827	0.000182	3.232	3.27E-08	***
Residuals	171	0.00963	0.000056			

Signif. codes: 0 '***' 0.001 '**' 0.01 '*' 0.05 '.' 0.1 ' ' 1

Table S7. Analysis of nFR variance contributed by individuals and compounds in the Berberine dataset

one-way ANOVA test, aov(nFR ~ Individual + Drug, data = data)

	Df	Sum Sq	Mean Sq	F value	Pr(>F)	Signif.
Individual	6	0.02443	0.004072	12.886	1.03E-10	***
Drug	17	0.00866	0.000509	1.612	0.0752	.
Residuals	99	0.03129	0.000316			

Signif. codes: 0 ‘***’ 0.001 ‘**’ 0.01 ‘*’ 0.05 ‘.’ 0.1 ‘ ’ 1

Table S8. Analysis of d_{ij} variance contributed by region, diagnosis and inflammation in the IBD dataset

```
Permutation test for adonis under reduced model
Terms added sequentially (first to last)
Permutation: free
Number of permutations: 999

Permutational Multivariate Analysis of Variance Using Bray-Curtis
Distance Matrix, adonis2(formula = data.dist ~ Region + Diagnosis +
Diagnosis:Inflammed, data = data.meta, permutations = 999)
```

	Df	SumOfSqs	R2	F	Pr(>F)	Signif.
Region	2	0.000306	0.01337	1.3252	0.186	
Diagnosis	2	0.002217	0.09692	9.6039	0.001	***
Diagnosis:Inflammed	2	0.000845	0.03693	3.6594	0.002	**
Residual	169	0.019503	0.85277			
Total	175	0.02287	1			

```
---
Signif. codes:  0 '***' 0.001 '**' 0.01 '*' 0.05 '.' 0.1 ' ' 1
```

Table S9. Analysis of d_{ij} variance contributed by individual and compounds in the RapidAIM dataset

```
Permutation test for adonis under reduced model
Terms added sequentially (first to last)
Permutation:
free
Number of permutations: 999

Permutational Multivariate Analysis of Variance Using Bray-
Curtis Distance Matrix, adonis2(formula = data.dist ~ Individual
+ Drug, data = data.meta, permutations = 999)
```

	Df	SumOfSqs	R2	F	Pr(>F)	Signif.
Individual	4	0.012101	0.38472	35.4164	0.001	***
Drug	43	0.004746	0.1509	1.2922	0.001	***
Residual	171	0.014607	0.46438			
Total	218	0.031454	1			

```
---
Signif. codes:  0 ‘***’ 0.001 ‘**’ 0.01 ‘*’ 0.05 ‘.’ 0.1 ‘ ’ 1
```

Table S10. Analysis of d_{ij} variance contributed by individuals and compounds in the Berberine dataset

```
Permutation test for adonis under reduced model
Terms added sequentially (first to last)
Permutation: free
Number of permutations: 999


Permutational Multivariate Analysis of Variance Using Bray-Curtis
Distance Matrix, adonis2(formula = data.dist ~ Individual + Drug, data =
data.meta, permutations = 999)
```

	Df	SumOfSqs	R2	F	Pr(>F)	Signif.
Individual	6	0.011612	0.22117	5.6832	0.001	***
Drug	17	0.007176	0.13669	1.2396	0.069	.
Residual	99	0.033714	0.64214			
Total	122	0.052503	1			

```
---
Signif. codes:  0 ‘***’ 0.001 ‘**’ 0.01 ‘*’ 0.05 ‘.’ 0.1 ‘ ’ 1
```
