# Supplementary material for: Revealing proteome-level functional redundancy in the human gut microbiome using ultra-deep metaproteomics
Source: Nat Commun. 2023 Jun 10;14:3428. doi: 10.1038/s41467-023-39149-2 (PMC10257714; doi:10.1038/s41467-023-39149-2)
Supplement: Supplementary file 3 — Reporting Summary [file 41467_2023_39149_MOESM3_ESM.pdf]

nature portfolio

Corresponding author(s): Yang-yu Liu, Daniel Figeys

Last updated by author(s): May 17, 2023

Reporting Summary

Nature Portfolio wishes to improve the reproducibility of the work that we publish. This form provides structure for consistency and transparency in reporting. For further information on Nature Portfolio policies, see our Editorial Policies and the Editorial Policy Checklist.

Statistics

For all statistical analyses, confirm that the following items are present in the figure legend, table legend, main text, or Methods section.

n/a	Confirmed	
☐	☒	The exact sample size (*n*) for each experimental group/condition, given as a discrete number and unit of measurement
☐	☒	A statement on whether measurements were taken from distinct samples or whether the same sample was measured repeatedly
☐	☒	The statistical test(s) used AND whether they are one- or two-sided<br>*Only common tests should be described solely by name; describe more complex techniques in the Methods section.*
☒	☐	A description of all covariates tested
☒	☐	A description of any assumptions or corrections, such as tests of normality and adjustment for multiple comparisons
☐	☒	A full description of the statistical parameters including central tendency (e.g. means) or other basic estimates (e.g. regression coefficient) AND variation (e.g. standard deviation) or associated estimates of uncertainty (e.g. confidence intervals)
☐	☒	For null hypothesis testing, the test statistic (e.g. *F*, *t*, *r*) with confidence intervals, effect sizes, degrees of freedom and *P* value noted<br>*Give P values as exact values whenever suitable.*
☒	☐	For Bayesian analysis, information on the choice of priors and Markov chain Monte Carlo settings
☒	☐	For hierarchical and complex designs, identification of the appropriate level for tests and full reporting of outcomes
☒	☐	Estimates of effect sizes (e.g. Cohen's *d*, Pearson's *r*), indicating how they were calculated

Our web collection on statistics for biologists contains articles on many of the points above.

Software and code

Policy information about availability of computer code

Data collection	LC-MS/MS data of the ultra-deep metaproteomics dataset was collected using Thermo Fisher Scientific Xcalibur™ Software (version 4.4).
Data analysis	Database search was performed following the MetaPro-IQ pipeline using X!Tandem (version 2015.12.15.2), MaxQuant (version 1.5.2.8) and MetaLab (version 1.2.0). Functional annotation to COG was performed using Diamond (version0.8.35). KEGG KOs were annotated using GhostKOALA (version 2.0). Computation of PCN, degree distribution, functional distance and functional redundancy were based on custom codes using Python (3.6.0) with packages pandas (version = 0.25.3) and numpy(version = 1.18.2). NODF values were computed using the R package RInSp. Jensen-Shannon divergence was calculated using the R package textmineR. Two-way ANOVA was performed using R function aov(). PERMANOVA tests were performed using R packages “vegan” and “BiodiversityR”. Kruskal-Wallis and Wilcoxon rank sum tests were performed using R functions kruskal.test() and wilcox.test(), respectively. Network incidence matrices, degree distributions, bar plots, box plots, and violin plots were visualized using the R package ggplot2. Unipartite networks were visualized using the R package igraph. Tripartite networks were visualized using the R package networkD3. Heatmaps were visualized using the R package pheatmap. Volcano plot was analyzed by MetaboAnalyst (version 4.0) under non-parametric test setting. The interactive webpage (https://shiny2.imetalab.ca/shiny/rstudio/PCN_visualizer/) for visualization of PCNs of metaproteomic datasets analyzed in this paper was created using the R packages shiny and shinydashboard. The Consumer-Resource Model model was implemented in Python 3.8 and Python packages Pandas, Numpy, Seaborn, and matplotlib.pyplot were used.

For manuscripts utilizing custom algorithms or software that are central to the research but not yet described in published literature, software must be made available to editors and reviewers. We strongly encourage code deposition in a community repository (e.g. GitHub). See the Nature Portfolio guidelines for submitting code & software for further information.

Data

Policy information about availability of data

All manuscripts must include a data availability statement. This statement should provide the following information, where applicable:

- Accession codes, unique identifiers, or web links for publicly available datasets
- A description of any restrictions on data availability
- For clinical datasets or third party data, please ensure that the statement adheres to our policy

The ultra-deep metaproteomics datasets were deposited to the ProteomeXchange Consortium (http://www.proteomexchange.org) via the PRIDE partner repository with the dataset identifier PXD027297. Database search outputs from the SISPROT (Zhang et al., 2017), RapidAIM (Li et al., 2020b), Berberine (Li et al., 2020a) and IBD (Zhang, Deeke, et al., 2018) studies have been previously deposited to the ProteomeXchange Consortium with the dataset identifiers PXD005619, PXD012724, PXD015934 and PXD007819, respectively. The four metagenomic datasets matching the ultra-deep metaproteomics datasets were obtained from the previous MetaPro-IQ study (Zhang et al., 2016), accessible from the NCBI sequence read archive (SRA) under the accession of SRP068619. Proteomics dataset of the cultured singles strain samples (Wang et al., 2022) has been previously deposited to the ProteomeXchange Consortium with the dataset identifier PXD037923. Source data are provided with this paper.

Human research participants

Policy information about studies involving human research participants and Sex and Gender in Research.

Reporting on sex and gender	For the ultra-deep metaproteomics analysis, intestinal aspirates samples were previously taken from the ascending colons of four individuals under 18 years of age and scheduled to undergo diagnostic colonoscopy (Zhang et al., 2016). In this study, we re-analyzed preserved aliquots of those samples using our ultra-deep approach to generate deep proteomic content network. Three were female donors and one was a male donor.
Population characteristics	See above. More detailed metadata about the research subjects are provided in Supplementary Table S1.
Recruitment	Eligible subjects were under 18 years of age and scheduled to undergo diagnostic colonoscopy.
Ethics oversight	The sample collection was approved by the Research Ethics Board of the Children's Hospital of Eastern Ontario (CHEO), Ottawa, ON, Canada. Written informed consent form was obtained from their parents. The study complies with all relevant ethical regulations for research with human participants.

Note that full information on the approval of the study protocol must also be provided in the manuscript.

Field-specific reporting

Please select the one below that is the best fit for your research. If you are not sure, read the appropriate sections before making your selection.

☒ Life sciences ☐ Behavioural & social sciences ☐ Ecological, evolutionary & environmental sciences

For a reference copy of the document with all sections, see nature.com/documents/nr-reporting-summary-flat.pdf

Life sciences study design

All studies must disclose on these points even when the disclosure is negative.

Sample size	This study includes a new ultra-deep metaproteomics dataset and four datasets from previously published works. (1) the ultra-deep metaproteomics analysis has a sample size of four (4 x 12 fractions = 48 raw files). The sample size of four is sufficient because here we aim to examine the network topology of individual microbiomes, and we did not aim to perform statistical analysis to compare between individuals/ groups. We show that the network topology in one individual microbiome can be reproduced in the other three microbiomes; (2) The SISPROT dataset (Zhang et al., 2017) has a sample size of 9; (3) The RapidAIM dataset (Li et al., 2020b) has a sample size of 219; (4) The Berberine dataset (Li et al., 2020a) has a sample size of 124; (5) The IBD dataset (Zhang, Deeke, et al., 2018) has a sample size of 176.
Data exclusions	No data was excluded from this study.
Replication	(1) the ultra-deep metaproteomics analysis has four biological replicates (four individual microbiomes), the replication successfully captured network topologies of different individual microbiomes; For other datasets obtained from literatures: (2) The SISPROT dataset has four biological replicates, and 2-3 technical replicates for each individual; (3) The RapidAIM dataset has five biological replicates for each drug treatment and control; (4) The Berberine dataset has seven biological replicates for each treatment and control; (5) The IBD dataset has 25 CD, 22 UC, and 24 non-IBD control subjects, providing biological replicates for each disease type/control group.
Randomization	For studying the network topology contribution to FR_p, four different randomization strategies were used to generate null networks. Each randomization was performed 10 times to demonstrate the reproducibility of the results. Randomization is not applicable to other part of the study since datasets were obtained from published literatures.

Blinding	Blinding is not applicable to this work, since for the experimental part, we aim to examine the network topology and redundancy of each individual microbiome, these microbiome are equally considered as biological replicates, there's no between-group comparisons.

Reporting for specific materials, systems and methods

We require information from authors about some types of materials, experimental systems and methods used in many studies. Here, indicate whether each material, system or method listed is relevant to your study. If you are not sure if a list item applies to your research, read the appropriate section before selecting a response.

Materials & experimental systems

n/a	Involved in the study
☒	☐ Antibodies
☒	☐ Eukaryotic cell lines
☒	☐ Palaeontology and archaeology
☒	☐ Animals and other organisms
☒	☐ Clinical data
☒	☐ Dual use research of concern

Methods

n/a	Involved in the study
☒	☐ ChIP-seq
☒	☐ Flow cytometry
☒	☐ MRI-based neuroimaging
